# Optimization of bioactives extraction from grape marc via a medium scale ambient temperature system and stability study

**DOI:** 10.3389/fnut.2022.1008457

**Published:** 2022-10-28

**Authors:** Aly Castillo, María Celeiro, Laura Rubio, Andrea Bañobre, Miguel Otero-Otero, Carmen Garcia-Jares, Marta Lores

**Affiliations:** ^1^Laboratorio de Investigación y Desarrollo de Soluciones Analíticas (LIDSA), Department of Analytical Chemistry, Nutrition, and Food Science, Universidade de Santiago de Compostela, Santiago de Compostela, Spain; ^2^CRETUS, Department of Analytical Chemistry, Nutrition, and Food Science, Universidade de Santiago de Compostela, Santiago de Compostela, Spain

**Keywords:** MSAT, polyphenols, antioxidant activity, grape marc, GRAS solvents, ethyl lactate, natural extracts, LC-MS/MS

## Abstract

A scalable procedure with minimum energy requirements, MSAT (Medium Scale Ambient Temperature), in combination with solvents generally recognized as safe (GRAS), has been optimized to obtain polyphenolic extracts from white grape (*Vitis vinifera*) marc. The solvents considered were propylene glycol (Pg), ethanol (Et), and ethyl lactate (Lc), as well as their respective hydro-organic mixtures. In a first approach, the operating parameters were optimized through a response surface matrix: extraction solvent volume (range 10–150 mL), marc mass (range 20–200 g) and marc/dispersant mass ratio (range 0.5–2 g⋅g^–1^), using the total polyphenol content (TPC) and the antioxidant activity (AA) of the extracts as response parameters. The highest TPC (5,918 mgGAE⋅L^–1^) and AA (44 mmolTE⋅L^–1^) values were obtained using 200 g marc and 100 mL solvent. Regarding the type of solvent, a better response was reached with Lc > Et > Pg > H_2_O obtaining a polyphenol concentration of 252 mg⋅L^–1^ for the hydro-organic isovolumetric ratio of ethyl lactate. In addition, the stability of the extracts was studied for 62 days. The effect of factors such as temperature, light exposure, and oxidative reactivity was evaluated. The bioactivity indices showed no changes with the storage conditions of the extracts in the first month of analysis, after which 75% of the antioxidant activity as the concentration of the polyphenolic profile (204 mg⋅L^–1^) remains. The absence of reactive oxygen and the cooling of the extract (4°C) were the most determining factors (*p* < 0.05) in modulating the stability of the total polyphenolic profile.

## Introduction

Within the main categories of agri-food exports of the European Union, wine production has dominated the basket of products in the last 5 years, with more than 165 million hectoliters generated annually (1). To meet this demand, more than 3.2 million hectares of vineyards are required, from which an average of 11 million tons of grapes are extracted each year, destined for winemaking purposes only (2). As a result of the winemaking process, around 25% in dry weight of this raw material is generated as a by-product, mainly comprised of grape skins, pips, and stems, called grape marc (3). This residual by-product causes an important handling and storage problem, where it’s discriminated and direct deposition in soils and groundwater leads to a potential phytotoxic. This is due to the release of high amounts of tannins and polyphenols, together with high oxygen consumption, which could have a negative impact on wildlife growth (4, 5). In contrast, the high content of bioactive compounds in grape marc, including both flavonoids and non-flavonoids, has shown important antioxidant and antimicrobial effects, being associated with the prevention of numerous diseases, including cardiovascular problems, neurodegenerative disorders such as Alzheimer’s, in addition to various forms of cancer (3, 6).

Currently, there are several extractive processes that seek to recover the bioactive compounds contained in grape marc (7). The most traditional are solid-liquid extraction by mechanical agitation and Soxhlet extraction, generally using ethanolic or methanolic solutions as solvents (8). The main challenges of these processes are the degradation of thermolabile compounds, long extraction time, toxicity, and environmental safety (9). Other techniques such as pressurized liquid extraction (PLE) (10), ultrasound assisted extraction (UAE) (11), microwave assisted extraction (MAE) (12), and supercritical fluid extraction (SFE) (5), are some of the current alternatives to industrial extractive processes. In general, these techniques face a complex trade-off between extractive efficiency and energy consumption, impacting on scale-up, such as the balance between optimal operating conditions (pressure and temperature) and extract integrity (13).

A scalable method, incorporating standard temperature and pressure conditions, MSAT (Medium Scale Ambient Temperature), has been widely applied in the extraction of polyphenolic compounds from plants (*Cytisus scoparius*) (14) and agro-industrial by-products (*Vaccinium corymbosum*) (15). Due to the scaling versatility of the process supported by a wide set of optimizable variables, and its combination with generally recognized as safe (GRAS) solvents such as ethyl lactate, glycols, ethanol, and aqueous solutions of mineral salts, it is an attractive technique for practical application purposes in different areas (16).

The synergistic activity provided by solvents in the generation of natural extracts is well known, not only in the overall obtaining of polyphenolic compounds, but also in their inherent bioactivity. Likewise, and contradictorily, isolation efforts toward individual analytes contained in plants are laborious, reducing their integral activity in fractionation, where the whole of their constituents is required to observe the biological effect (17). Moreover, the versatility of obtaining a ready-to-use ingredient in the liquid state provides a range of advantages in relation to diffusion, dosage, and application. However, a fundamental part of natural additive formulations is the evaluation of their chemical stability as a finished product during the storage period, which is of particular importance for liquid products (16). The stability of the compounds that make up a nutraceutical formulation can be affected due to changes in storage conditions resulting in the modification of their nutritional value (e.g., antioxidant capacity, composition, and bioavailability) (18). Polyphenols present unsaturated bonds and a high antioxidant capacity, which makes them sensitive to heat, pH variations, light, and the presence of oxygen (19). At the same time, they show a heterogeneous degradation profile, being dependent both on their phenolic nature and on the operational conditions under which they are obtained. This highlights the need to know and control both the extraction process and the stability and preservation conditions of each specific extract.

Therefore, this work performs a comprehensive analysis of the production of bioactive extracts from grape marc using the MSAT extraction technique. Based on the optimization study of the operational parameters involved in the extractive process, the total polyphenolic content (TPC) and antioxidant activity (AA) of the extract were maximized. Also, the affinity selected of the GRAS solvents: ethyl lactate, ethanol, and propylene glycol, as well as their hydro-organic mixtures, toward the main phenolic families contained in the marc, was evaluated. In addition, the stability of the extracts generated when stored under various conditions of temperature, light and oxidative exposure was analyzed by means of a 62-day periodic test.

## Materials and methods

### Standard and reagents

The standards used for the quantification of bioactivities as well as the individual characterization of the main polyphenolic compounds contained in the white grape extract, with their CAS numbers, purity, and suppliers are summarized in Supplementary Table 1. The solvents used for the extraction process were ethanol, ethyl lactate, propylene glycol, and ultrapure water of MS grade from Scharlab (Barcelona, Spain). MS-grade methanol obtained from Sigma-Aldrich Chemie GmbH (Steinheim, Germany) and formic acid from Merck (Darmstadt, Germany), were used to prepare the mobile phases for chromatographic analysis.

### Grape marc

In this work, the marc for the 2021 harvest was obtained directly from Galician vineyards, specifically from Albariño white grapes. The grapes were cultivated in the Rías Baixas denomination of origin, in the subzone of O Salnés, at the Mar de Frades winery. Once the grapes were pressed in the winery, the marc was collected and placed in food-grade bags (20 cm × 20 cm) hermetically sealed for freezing (–20°C). The marc, which has a moisture content of 65.37% and an acid pH of 4.19, was kept in these conditions until the time of processing to obtain the extracts.

### Medium-scale ambient temperature extraction

Frozen marc was weighed according to design specifications and crushed without pre-treatment in a food grinder (Moulinex, France) until an average particle diameter of 5 mm was obtained. The marc was then dispersed with SiO_2_ (particle size 0.707 mm) using a mortar and pestle for 5 min. MSAT extraction was carried out on an Afora V-53721 glass column (23 cm × 50 mm Ø) with a 0-pore filter plate (160–250 μm) containing 1 g of SiO_2_ layer at the bottom. Then, the mixture of disrupted marc and SiO_2_ was transferred to the column and compacted. Finally, the extract was eluted with the different volumes and solvents considered in the study by maintaining a controlled extractive flow of 2 mL⋅min^–1^.

### Total polyphenol content index

The total polyphenolic content (TPC) index of grape marc extracts was determined by the Folin-Ciocalteu method following Zhang’s guidelines for microtitration in 96-well plates (20). Briefly, a total of 20 μL of pure extract without further modifications is diluted and subsequently mixed with 100 μL of Folin-Ciocalteu reagent (1:10, v/v) and 80 μL of sodium carbonate solution (7.5 g⋅L^–1^). The mixture was shaken and isolated in the dark for 30 min and then measured at 760 nm in a microplate reader (BMG LABTECH, Ortenberg, Germany). To express the TPC index, calibration curves of gallic acid covering a concentration range of 20–160 mg⋅L^–1^ (0.200–0.800 absorbance unit [AU]) were employed. TPC was expressed as milligrams of gallic acid equivalent per liter of extract (mgGAE⋅L^–1^).

### Antioxidant activity

The antioxidant activity (AA) of the extracts, as well as their mean inhibitory concentration IC50, were determined using the DPPH reagent following the method described by Symes et al. (21). Briefly, 100 μL of the extracts at eight different concentration levels were placed in a 96-well plate and mixed with 100 μL of DPPH reagent prepared in methanol. The mixture was kept in the dark for 10 min and the measurement was performed at 515 nm. To express the AA, a calibration curve of Trolox in the range of 3–31 mg⋅L^–1^ (0.200–0.800 AU) was employed. The AA was represented as millimoles Trolox equivalent per liter of extract (mmolTE⋅L^–1^). The half inhibitory concentration (IC50) of the samples was also measured and referred to the total polyphenolic content (mgGAE⋅L^–1^) present in the extract.

### Reducing sugars

The total reducing sugar content of the extracts was determined by the 3,5-dinitrosalicylic acid (DNS) method following the method of Gonçalves et al. (22) with slight modifications. Briefly, 25 μL of the extract was mixed with 25 μL of DNS reagent. The reaction takes place in a bath at 100°C for 5 min. The measurement was performed at 540 nm. To express the total content of reducing sugars, a calibration curve of glucose was used in the range of 0.5–2 mg⋅L^–1^ (0.200–0.800 AU). Results were represented as mg glucose equivalent (mg GLE) per liter of extract.

### LC-MS/MS analysis

The quantification of the polyphenols in the extracts was performed by LC-MS/MS using a Thermo Scientific (San Jose, CA, USA) instrument based on a TSQ Quantum Ultra™ triple quadrupole mass spectrometer equipped with a heated electrospray ionization (HESI) source, and an Accela Open autosampler with a 20 μL loop. Optimal instrumental conditions were previously optimized by Celeiro et al. (23). The chromatographic separation was performed employing a Kinetex C18 column (100 mm × 2.1 mm × 100 Å) obtained from Phenomenex (Torrance, CA, USA). The mobile phase was composed of water (A) and methanol (B), both with 0.1% formic acid. The chromatographic gradient was 5% B to 90% B in 11 min and kept constant for 3 min. Initial conditions were achieved in 6 min. Injection volume was 10 μL, with a flow rate of 0.2 mL⋅min^–1^, and column temperature was set at 50°C. Compound identification and detection were performed by selected reaction monitoring (SRM) working simultaneously in both negative and positive mode, monitoring two or three MS/MS transitions for each compound. The MS/MS parameters for all studied compounds were optimized by individual direct infusion and the most abundant collision-induced fragments were considered for quantification (Supplementary Table 2). Other HESI source parameters were the spray voltage: 3,000 V, vaporizer temperature: 350°C, sheath gas pressure: 35 au (arbitrary units), and ion sweep and auxiliar gas pressure: 0 and 10 au, respectively, and the capillary temperature: 320°C. The system was operated by Xcalibur 2.2. and Trace Finder 3.1. software.

### Statistical analysis

Analyses were performed at least in triplicate and results were expressed as mean ± standard deviation. The optimization of the extraction procedure was carried out by a composite central response surface design evaluating the quadratic action of the considered factors. The solvent study was analyzed through a general linear one-way analysis of variance (ANOVA), comparing the individual action in contrast to the control group (100% water) by least significance difference (LSD) test. For the evaluation of the effect of storage parameters on the stability of the extract, a general linear ANOVA was performed using the Dunnett test for the comparative analysis with the control groups. The statistical analysis was performed using Minitab 22.1.1 software (Pennsylvania, USA). 3D and 2D response surface plots were generated using OriginPro, Version 2021 (OriginLab Corporation, Northampton, MA, USA).

## Results and discussion

### Medium scale ambient temperature system operational optimization

In a first approach, an operational range was delimited considering the process variables: extract volume, marc mass, and dispersant/marc ratio, keeping fixed the extractant solvent (ethanol-water 50:50) and SiO_2_ as disruptor (Table 1). The ethanol-water mixture was initially selected due to its high capacity for the extraction of various phenolic compounds from agro-industrial residues (24, 25), resulting in extracts characterized by a higher antioxidant activity than extracts obtained with pure water or ethanol (26). As for the dispersant material, SiO_2_ from sea sand was selected over other materials such as Florisil or diatomaceous earth (DE). The limited size range, typically less than 150 μm for DE, as well as higher costs for Florisil decreased the feasibility of the optimization process and subsequent scale-up.

**TABLE 1:** Extraction parameters and factor levels used in the surface design for medium scale ambient temperature (MSAT) extraction.

Parameters	Legend	Fixed	Low	High
Marc (g)	A	–	20	100
Extraction solvent (mL)	B	Ethanol-water 50:50	10	250
Dispersant/marc (g/g)	C	Dispersant: SiO_2_	0.5	2

For the analysis of operational factors, a composite center response surface matrix (RSM) design divided into three orthogonal blocks was used to provide a measure of operational stability throughout the tests. Table 2 summarizes the design matrix comprising 20 trials bounded by eight hub area points, six axial points, four hub center points, and two axial center points. The TPC and the AA of the extracts were considered as the response variables to evaluate the results of the design.

**TABLE 2:** Design matrix and results obtained for bioactive indexes total polyphenol content (TPC) and antioxidant activity (AA).

Assay	Type	Block	A	B	C	TPC (mgGAE⋅L^–^^1^)	AA (mmolTE⋅L^–^^1^)
1	Central	2	60	130	1.25	3,016	23.1
2	Central	2	60	130	1.25	2,998	19.0
3	Cube	2	36	57	1.71	3,679	27.0
4	Cube	2	36	203	0.79	2,550	19.5
5	Cube	2	84	203	1.71	2,677	16.7
6	Cube	2	84	57	0.79	3,128	18.3
7	Central	1	60	130	1.25	2,793	13.2
8	Cube	1	84	57	1.71	3,727	21.5
9	Central	1	60	130	1.25	2,829	26.7
10	Cube	1	36	203	1.71	2,068	18.2
11	Cube	1	84	203	0.79	2,271	17.9
12	Cube	1	36	57	0.79	1,634	12.5
13	Central	3	60	130	1.25	2,319	17.7
14	Central	3	60	130	1.25	2,791	23.1
15	Axial	3	60	130	2.00	3,714	30.6
16	Axial	3	60	10	1.25	3,304	25.8
17	Axial	3	60	250	1.25	1,642	11.9
18	Axial	3	60	130	0.50	2,321	20.5
19	Axial	3	20	130	1.25	2,009	18.8
20	Axial	3	100	130	1.25	3,508	28.1

A, Marc mass (g); B, extract volume (mL); C, dispersant/Marc ratio.

The TPC values show clear differences among the design experiments. All three factors were statistically significant: the amount of marc (A, *p* = 0.009), the volume of extraction solvent (B, *p* = 0.003) and the dispersant/marc ratio (C, *p* = 0.005), as well as the ratio between factors B and C (*p* = 0.023). Antioxidant activity followed the same behavior as TPC, although for this parameter the design factors were not statistically significant.

**FIGURE 1:**
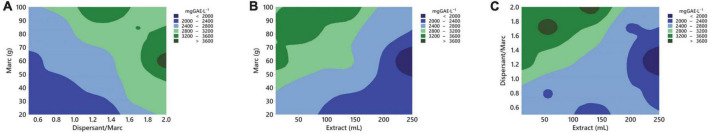
Response surface matrix for total polyphenolic content (TPC, mgGAE⋅L^–1^) by modifications in the operational variables of the medium scale ambient temperature (MSAT) extractive process. **(A)** Dispersant/marc × marc. **(B)** Extraction solvent × marc. **(C)** Extraction solvent × dispersant/marc.

Figure 1 shows the response surface plots obtained for TPC, in which a progressive trend of TPC is observed, from 1,600 to 3,800 mgGAE⋅L^–1^, with increasing marc mass and decreasing solvent volume, as well as with the marc-dispersant ratio. Values close to the lower limit are obtained by applying MSAT to sources rich in polyphenols, mainly anthocyanins and procyanidins, such as blueberries, generating TPC in the order of 1,455 mgGAE⋅L^–1^ (18). By applying other processes such as ultrasound-assisted extraction (UAE), liquid bioactive extracts have been generated from bagasse from organic sources such as carrots with TPCs in the order of 1,903.2–2,754.9 mgGAE⋅L^–1^ (19).

At the same time, direct comparison of the obtained TPC range (1,634–3,727 mgGAE⋅L^–1^) with other extractive processes from white grape marc is complex, due to a general solid extract production approach, where the extractive yield is referred to the initial marc mass content (mgGAE⋅g^–1^). Values have been reported for ethanolic extracts via UAE from Merlot grape marc from Krèedin grape variety, in the range of 806–2,800 mGAE⋅L^–1^ (20). Although a different polyphenolic content is expected due to being from the red winemaking process, such values are obtained by processes with higher energy requirements in relation to the MSAT technique, as well as mass values below 2 g.

Another aspect that stands out in Figure 1 is that the area delimiting the maximum sectors is partially located at the extremes of the experimental domain considered. Thus, the addition of new experiments was proposed to extend the experimental design matrix. These experiments are delimited through an optimization algorithm using predictive tests to increase the polyphenolic content and antioxidant activity while respecting the operational limits of the extractive column.

**FIGURE 2:**
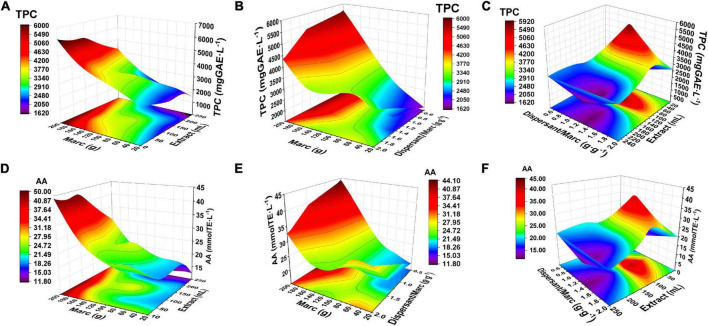
Statistical optimization of operational variables and their impact on total polyphenol content (TPC) and antioxidant activity (AA) bioactive indicators in the medium scale ambient temperature (MSAT) extractive process through the expanded experimental domain using response surface matrix (RSM). For TPC response: **(A)** Extraction solvent × marc. **(B)** Dispersant/marc × marc. **(C)** Extraction solvent × dispersant/marc. For the AA response: **(D)** Extraction solvent × marc. **(E)** Dispersant/marc × marc. **(F)** Extraction solvent × dispersant/marc.

Table 3 shows the results obtained with the additional points of the design, confirming that the TPC and AA values increase with the mass of marc extracted. In general, good agreement was obtained between the predicted and measured values, although in experiment 22 the measured TPC and AA values were higher than the predicted values. When incorporating the new experiments, the response surface plots (Figure 2) revealed a significant interaction between marc mass and dispersant-marc ratio (AC interaction) with *p* = 0.005 and *p* = 0.024 for TPC and AA, respectively. The optimum working zone corresponds to the values of 200 g of marc, 100 mL of extracting solvent and 1.25 marc-dispersant ratio, obtaining a maximum TPC of 5,918 mgGAE⋅L^–1^. This value represents a high level in contrast to other extractive procedures, such as enzymatic processes for the extraction of phenols from the marc of white grapes, where TPCs of the order of 1,272–2,659 mgGAE⋅L^–1^ are obtained (21), showing higher MSAT values (4.6 and 2.2 times, respectively).

**TABLE 3:** Additional experiments to expand the experimental design matrix and prediction results for total polyphenol content (TPC) and antioxidant activity (AA).

Assay	A	B	C	Predicted TPC (mgGAE⋅L^–^^1^)	Real TPC (mgGAE⋅L^–^^1^)	Predicted AA (mmolTE⋅L^–^^1^)	Real AA (mmolTE⋅L^–^^1^)
21	150	150	2	3691	3393	22.6	24.7
22	200	100	1.25	4528	5918	34.1	44.0
23	165	75	1.70	4937	4140	29.6	28.2

A, Marc mass (g); B, extract volume (mL); C, dispersant/Marc ratio.

### Solvent selection

Once the operational parameters were optimized, a study was carried out to select the best solvent considering only GRAS solvents: ethyl lactate, ethanol and propylene glycol mixed with water in different percentages (25, 50, 75, and 100%). To compare the extraction efficiency, a general linear ANOVA was used to determine the individual effect by means of LSD comparative analysis, considering 100% water as the control solvent. The analysis of the results considered: the content of reducing sugars and the acidity of the extracts; the bioactive profile of the extracts measured by TPC, AA, and IC50; and the polyphenolic profiles, evaluating 11 different polyphenols.

#### Total reducing sugar content and extract acidity

The industrial production of bioactive extracts from plants and agricultural by-products usually generates liquid products. However, a total or partial drying process of the extracts is often implemented to reduce their volume and/or to improve their handling and application properties (27). Spray drying and lyophilization are the most used techniques to produce solid natural extracts. Other alternatives to solvent removal such as vacuum evaporation and drying by N_2_ current have been successfully applied to ethanolic and ethyl lactate natural extracts at temperatures not higher than 100°C (28). It is important to consider that when these techniques are used in food media rich in sugars and acids, they present compaction problems, since the glass transition temperature (20°C) is exceeded, when a hard, solid, and amorphous sugar undergoes a transformation to a liquid, soft and gummy phase, generating a semi-solid state that is not very manageable (29). In addition, factors such as acidity play a role in modulating the bioactivity of the extracts, with a greater degradation of the polyphenolic content in basic media (30). Since the aim is to obtain a versatile extract that can be used for solid-state production while maintaining maximum bioactivity, the selected response factors were the content in reducing sugars and the pH of the extract. The results are shown in Table 4.

**TABLE 4:** Grape marc extraction solvent selection using the medium scale ambient temperature (MSAT) extractive technique: effect on reducing sugars content and pH.

Solvent	Rate	Reducing sugars (g⋅L^–^^1^)	pH
Water	100	51.26	4.31
Ethanol	25	46.51	4.44^H^
	50	53.03	4.62^H^
	75	47.84	4.72^H^
	100	40.00	4.96^H^
Ethyl Lactate	25	44.86	4.22
	50	44.07	4.11^L^
	75	41.17	4.62^H^
	100	34.91^L^	4.73^H^
Propylene glycol	25	42.88	4.42
	50	51.47	4.47^H^
	75	46.09	4.58^H^
	100	48.20	4.63^H^
LSD Test		*p* > 0.05	*p* < 0.001

^H^Upper significant value. ^L^Lower significant value.

No significant effect (*p* > 0.05) was evidenced in the extraction of reducing sugars because of the modification of the solvents considered. However, the analysis shows a decrease of more than 30% of the total sugar content when pure ethyl lactate is used as extractant. The selectivity of ethyl lactate toward bioactive compounds and its low affinity toward carbohydrates such as glucose and sucrose has been reported in natural algal extracts, where less than 1% of these compounds are obtained in relation to other solvents such as ethanol, methanol, and acetone (31). As for the pH, it ranged from 4.11 to 4.96, with lower values, in general, the lower the water content in the solvent mixture. The 50:50 ethyl lactate-water mixture resulted in extracts with the highest acidity.

#### Bioactive profile of the extracts: Total polyphenol content, antioxidant activity, and IC50

**FIGURE 3:**
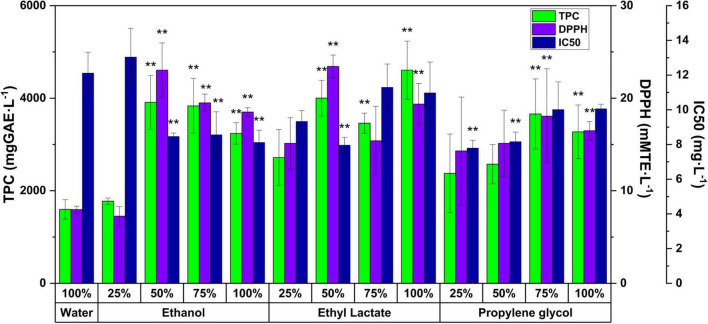
Response of the bioactive indicators antioxidant activity (AA), total polyphenol content (TPC), and IC50 to modifications in the proportions of the extractive solvents. **Significant value.

The bioactivity of natural extracts is strongly conditioned by the type of extractant solvent and its capacity for the extraction of bioactive compounds with different polarities (32). Ethanolic-based solvents have been widely used demonstrating a high antioxidant potential. Other solvents such as propylene glycol, widely used in the feed field, and ethyl lactate, used in the food industry, have been much less explored for the extraction of natural bioactive compounds (33, 34). For the first time here is reported a global analysis of white grape marc that incorporates the evaluation of these three GRAS solvents in line with a minimum requirements technique such as MSAT system. In this work, the effect of ethanol, ethyl lactate, and propylene glycol and their mixtures with water on widely recognized bioactivity indexes: TPC, AA, and mean inhibition index (IC50) has been evaluated. Figure 3 shows the results obtained. In general, it is evident that in all cases there is a correlation between the TPC and the AA, indicating that the polyphenolic compounds are the main causes of the free radical inhibitory power of the white grape marc extract. The mixture of water and 50% ethyl lactate as opposed to the pure organic solvent, obtained the best set of parameters: DPPH (23.4 mmolTE-L^–1^) and IC50 (8.0 mg-L^–1^), as an analogous TPC (4,003 mgGAE-L^–1^). A similar behavior was evidenced by Lores et al., using ethyl lactate for the extraction of bioactive compounds from the plant *Cytisus scoparius*, attributing such performance to the different polarities of the solvents which allow the extraction of a wider range of compounds (14). Ethanol at the same ratio of 50% in water generated very similar results. The figure also shows a significant effect on the TPC when using solvents with a lower proportion of water. The maximum TPC value (4,606 mgGAE⋅L^–1^) of the extract obtained with pure ethyl lactate stands out. Also, it is relevant the IC50 value obtained with the lowest proportion of propylene glycol in water (25%).

#### Polyphenolic profiles

The extracts obtained with the different solvents tested showed diverse polyphenolic profiles considering the 11 phenolic compounds determined by LC-MS/MS (Supplementary Figure 1). When the sum of the concentrations of the individual compounds is evaluated (Supplementary Table 3), the best solvents are ethanol and ethyl lactate 50% in water, followed in efficiency by the same pure solvents. In general, ethanol presents high affinity for most of the polyphenolic families. Ethanol/water (50:50, v/v) was significantly superior for the extraction of procyanidins (134 mg⋅L^–1^). The affinity of ethanol for these flavonoids, which account for more than 50% of the polyphenols in the extract, makes it the solvent with the highest overall extraction efficiency. Below ethanol, with less than 5% difference, ethyl lactate was significantly more selective for quercetin (5.4 mg⋅L^–1^), being more than 60% higher than the other solvents. The similarity of these two solvents toward the extraction of bioactive compounds has been evidenced in polyphenolic families, as well as in terpenes (35) and carotenoids (36), where in the latter, ethyl lactate not only generated higher recoveries but also a greater modulation to the degradation of these thermolabile and photosensitive compounds (34). The extracts obtained with propylene glycol did not show significant differences on the total polyphenol content, due to their selectivity for the polyphenols found in lower concentrations in the extracts, especially caffeic, epigallocatechin gallate, and gallic acid.

The differences in the selectivity of the solvents tested for obtaining bioactive extracts from white marc generate the need for a compromise in the selection of an extraction solvent to obtain an extract that can be dried for production as a solid while maintaining the best combination of bioactive parameters, as well as a comprehensive polyphenolic profile. Since 50% ethyl lactate in water generated the lowest concentration of total sugar content, it is the potential candidate to produce solid extracts, maintaining high TPC, AA, and IC50 values, and a balanced polyphenolic profile. In turn, its application in the extractive process of grape marc as a food grade, non-toxic and non-carcinogenic solvent, approved for use in food and pharmaceuticals (33), results in an attractive and current approach involving a low-energy extraction (MSATs) and GRAS solvents. Therefore, this solvent was selected to continue the study, evaluating the stability of the extracts.

### Stability of extracts

#### Bioactive profile and acidity stability

Table 5 shows the effects of the factors time, temperature, light exposure, and reactivity with oxygen on the main bioactive indices TPC, AA, IC50, and acidity of the extract. The statistical significance of the factors considered was determined by a matrix of 24 trials through a general linear ANOVA. The individual behavior of the factors was analyzed by applying Dunnett comparative test, considering the original extract (day 0) as a control group, as well as the minimum storage conditions (20°C, absence of light, and oxidative protection). The results of the statistical analysis revealed that both TPC and IC50 did not show significant variation under the different storage conditions. However, AA shows a slight modulation of 3.6% when oxygen is removed from the storage medium. In addition, the extract shows a reduced acidification (*p* < 0.05) of about 8% from the initial value when stored at 20°C, with only a 5 or 6% decrease in pH at temperatures of –20 and 4°C, respectively.

**TABLE 5:** Stability of bioactive parameters total polyphenol content (TPC), antioxidant activity (AA), and IC50, and pH of white grape marc extract in 50% ethyl lactate extractant to modification in storage conditions.

Factor	Values	TPC (mgGAE⋅L^–^^1^)	AA (mmolTE⋅L^–^^1^)	IC50 (mgGAE⋅L^–^^1^)	pH
Day	0	3,677	19.0	7.6	4.65
	2	3,518	16.1	13.2^H^	4.52
	4	3,815	17.6	10.0	4.53
	8	5,028^H^	19.9	14.9^H^	4.49
	20	3,681	13.8^L^	13.6^H^	4.37
	34	3,286	14.1^L^	15.8^H^	4.32^L^
	62	3,479	12.9^L^	15.4^H^	4.14^L^
*p*-value		<0.001	<0.001	<0.001	<0.001
Light	No	3,802	16.2	13.0	4.43
	Yes	3,764	16.2	12.8	4.43
*p*-value		>0.05	>0.05	>0.05	>0.05
Nitrogen	No	3,743	15.9	13.1	4.43
	Yes	3,823	16.5^H^	12.8	4.43
*p*-value		>0.05	0.030	>0.05	>0.05
Temperature (°C)	–20	3,780	16.3	13.1	4.55^H^
	4	3,802	16.4	12.7	4.468^H^
	20	3,768	16.0	13.0	4.274
*p*-value		>0.05	>0.05	>0.05	<0.001

^H^Upper significant value. ^L^Lower significant value.

The extract shows a significant increase (*p* < 0.05) of more than 35% of the TPC on the 8th day of analysis, decreasing to the initial values for the following points of the study. However, this increase for TPC does not show a correlation with either AA or IC50. In this respect, there are previous references of similar behavior in stability studies of polyphenolic compounds, showing singular increases in the concentration of some of these compounds due to the degradation of other phenolic chains (37). A heterogeneous response in the reactivity of the Folin-Ciocalteu method to different compounds has also been demonstrated, showing that polyphenols such as quercetin, gallic acid, ellagic acid, and ferulic acid, at the same concentration, generate a 50% higher reactivity in the presence of Folin’s reagent with respect to larger compounds such as rutin (38). Thus, the point increase in TPC obtained in the stability study could be due to the specific fragmentation of the large phenolic chains into smaller but more Folin-reactive compounds.

#### Individual polyphenolic profile stability

The evolution of the 11 polyphenolic compounds characterized in the extract under different storage conditions is summarized in Supplementary Table 4. The individual polyphenolic profiles during the 62 days considered and their main degradation modifiers are shown in Supplementary Figure 2.

The choice of 11 polyphenolic markers as indicators of extract stability under light, oxidative, and temperature effects make this evaluation a pioneering analysis of grape marc extracts, giving a better understanding of the interactions between these bioactive compounds.

In general, a significant fluctuation in the profiles is observed, where the relationship between degradation and the formation of smaller polyphenolic compounds from others with larger structures keeps the total content stable until the 8th day. Quercetin-3-glucuronide and caftaric acid are more stable, retaining up to 60% of their initial concentration during 34 days of storage. In contrast, larger polyphenolic compounds, such as rutin, and procyanidins, show a rapid degradation of around 55% from the 4th day onward. Although the absence of oxygen improves the preservation of procyanidins, the degenerative effect produced by a higher storage temperature has a greater impact on the preservation of these compounds. This behavior has been previously evidenced by Rohr et al., considering these compounds as the most unstable natural phenolic chains, with the capacity to spontaneously oxidize in the presence of oxygen, as well as their enzymatic degradation by interaction with polyphenol oxidase (39). In contrast, only temperature generates a degenerative effect in the case of rutin, decreasing its concentration by up to 63%. The thermolability of rutin has been discussed by Murakami et al., showing a higher sensitivity of this flavonoid in contrast to phenolic acids such as chlorogenic acid (40).

The polyphenolic compounds quercetin and gallic acid, together show a similar pattern of evolution, increasing in concentration by up to 200% over the 62 days of storage, being favored by higher temperatures (Supplementary Table 4). This pattern of behavior, analogous to that observed in the analysis of the TPC, is generated by the decomposition of the larger structures containing them. In this regard, it is well known that hydrolyzable tannins from glucose esters of gallic acid generate gallic acid as well as its carbohydrate substituents (41). Similarly, Supplementary Figure 2 shows a decrease in the concentration of quercetin-3-glucoside and quercetin-3-glucuronide acids that is proportional to the increase in quercetin content.

## Conclusion

The aim of this work was to obtain polyphenolic extracts from grape marc by means of an extractive method with minimum energy requirements MSAT (Medium Scale Ambient Temperature) system, using solvents generally recognized as safe (GRAS). The set of operational parameters including extractive volume (100 mL), marc mass (200 g) and its ratio with a dispersant (1.25) were efficiently optimized by applying a response surface matrix (RSM) design, obtaining maximum TPC (5,918 mgGAE⋅L^–1^) and AA (44 mmolTE⋅L^–1^) values. The robustness in the response of the operational variables together with a high correlation between the predicted response and the experimental values obtained, demonstrate the versatility of MSATs for the development of process scaling-up. Ethyl lactate was the solvent that provided the best extraction efficiency, with its 50:50 water ratio yielding extracts with the best response for bioactivity parameters, and the highest extraction of the flavonols, quercetin, and their glycoside derivatives. Also, an ethanolic extract was obtained, more enriched in flavanols and procyanidins, as well as a glycolic extract with a selective efficacy for caftaric and gallic acids. A study of the stability of the extract during 2 months of storage showed that it retains 62% of its antioxidant activity and keeps its total polyphenolic content unchanged. Thermal and light effects, as well as the presence of oxygen in the storage of the extract, did not generate significant overall changes in the bioactive properties.

## Data availability statement

The original contributions presented in the study are included in the article/Supplementary material, further inquiries can be directed to the corresponding author/s.

## Author contributions

ML and CG-J: conceptualization, funding acquisition, and methodology. AC: software and visualization. AB, MO-O, and AC: validation. AC, LR, and MC: formal analysis. AB and AC: investigation and writing—original draft preparation. ML: resources. AC and MO-O: data curation. AC, LR, MC, ML, and CG-J: writing—review and editing. MC, ML, and CG-J: supervision. AC, ML, and CG-J: project administration. All authors have read and agreed to the published version of the manuscript.
