# Supplementary material for: Optimization of bioactives extraction from grape marc via a medium scale ambient temperature system and stability study
Source: Front Nutr. 2022 Oct 28;9:1008457. doi: 10.3389/fnut.2022.1008457 (PMC9650278; doi:10.3389/fnut.2022.1008457)
Supplement: Supplementary file 1 [file Data_Sheet_1.docx]

Supplementary Material

**Supplementary Table 1** Studied compounds in white grape marc and standards for analysis. CAS numbers, purity, and suppliers

| **Name** | **Formula** | **Purity (%)** | **Company** | **CAS** |
| --- | --- | --- | --- | --- |
| **Polyphenols** | | | | |
| Gallic acid | C_7_H_6_O_5_ | 99.9 | ^a^SIGMA | 149-91-7 |
| Caftaric acid | C_13_H_12_O_9_ | 99.3 | ^a^SIGMA | 67879-58-7 |
| Procyanidin B1 | C_30_H_26_O_12_ | 96.7 | ^b^EXTRAS | 20315-25-7 |
| Catechin | C_15_H_14_O_6_ | 98.0 | ^a^SIGMA | 18829-70-4 |
| Epicatechin | C_15_H_14_O_6_ | 90.0 | ^a^SIGMA | 490-46-0 |
| Epigallocatechin gallate | C_22_H_18_O_11_ | 99.1 | ^a^SIGMA | 989-51-5 |
| Epicatechin gallate | C_22_H_18_O_10_ | 98.0 | ^a^SIGMA | 1257-08-5 |
| Quercetin-3-glucuronide | C_21_H_18_O_13_ | 98.5 | ^a^SIGMA | 27253-19-6 |
| Rutin | C_27_H_30_O_16_ | 99.1 | ^a^SIGMA | 115888-40-9 |
| Quercetin-3-glucoside | C_21_H_20_O_12_ | 98.0 | ^a^SIGMA | 21637-25-2 |
| Quercetin | C_15_H_10_O_7_ | 96.0 | ^a^SIGMA | 117-39-5 |
| **Other standards** | | | | |
| DPPH | C_18_H_12_N_5_O_6_ | 99.2 | ^c^TCI | 1898-66-4 |
| Folin reagent | C_10_H_5_NaO_5_S | - | ^a^SIGMA | 521-24-4 |
| 3,5-Dinitrosalicylic acid (DNS) | C_7_H_4_N_2_O_7_ | 99.9 | ^e^ALFA | 609-99-4 |
| Glucose | C₆H₁₂O₆ | 98.7 | ^c^TCI | 50-99-7 |
| Trolox | C_14_H_18_O_4_ | 98.5 | ^a^SIGMA | 53188-07-1 |
| Sodium carbonate | Na_2_CO_3_ | 99.7 | ^d^PANREAC | 497-19-8 |

^a^Sigma Aldrich GmbH (Steinheim, Germany), ^b^Extrasynthese (Genay, France), ^c^TCI (Tokyo Chemical Industry) (Tokyo, Japan), ^d^Panreac AppliChem (Barcelona, Spain), ^e^Alpha Aesar (Kandel, Germany).

**Supplementary Table 2** Linear range, coefficients of determination (R^2^), retention time (Rt) and MS/MS transitions for the identified polyphenols in the concentrate of grape marc extracts.

| **Polyphenols** | **Rt**  **(min)** | **Formula** | **Precursor Ion**  **(m/z)** | **Product Ion**  **(m/z)** | **Collision Energy**  **(eV)** | **Linear Range**  **(mg·L^-1)^** | **R^2^** |
| --- | --- | --- | --- | --- | --- | --- | --- |
| Gallic acid | 2.35 | C_7_H_6_O_5_ | 169.020 | 125.037 | 17 | 5-10 | 0.9965 |
| Caftaric acid | 4.41 | C_13_H_12_O_9_ | 310.958 | 178.968 | 17 | 1-5 | 0.9926 |
| Procyanidin B1 | 5.30 | C_30_H_26_O_12_ | 577.033 | 407.066 | 26 | 5-10 | 0.9958 |
| Catechin | 5.34 | C_15_H_14_O_6_ | 289.006 | 245.020 | 17 | 5-10 | 0.9869 |
| Epicatechin | 6.50 | C_15_H_14_O_6_ | 289.006 | 245.020 | 17 | 5-10 | 0.9949 |
| Epigallocatechin gallate | 6.80 | C_22_H_18_O_11_ | 457.151 | 169.059 | 21 | 5-10 | 0.9793 |
| Epicatechin gallate | 7.29 | C_22_H_18_O_10_ | 441.133 | 289.126 | 20 | 1-5 | 0.9807 |
| Quercetin-3-glucuronide | 9.54 | C_21_H_18_O_13_ | 479.090 | 302.966 | 18 | 5-10 | 0.9955 |
| Rutin | 9.72 | C_27_H_30_O_16_ | 609.182 | 270.917 | 56 | 5-10 | 0.9828 |
| Quercetin-3-glucoside | 9.75 | C_21_H_20_O_12_ | 465.076 | 302.971 | 14 | 5-10 | 0.9924 |
| Quercetin | 11.83 | C_15_H_10_O_7_ | 303.098 | 229.106 | 28 | 5-10 | 0.9863 |

**Supplementary Table 3**  Polyphenolic profile of white grape extract towards solvents: Ethanol (Et), Ethyl lactate (Lc), Propylene glycol (Pg) and ratios (25, 50, 75, 100) to water (W).

| **Concentration (mg·L-1)** | | | | | | | | | | | | |
| --- | --- | --- | --- | --- | --- | --- | --- | --- | --- | --- | --- | --- |
| **Solv.** | **Gallic A.** | **Catechin** | **Epi.** | **Epigallo. g.** | **Epica. g.** | **ΣProcyan.**  **(B1+B2+C1)** | **Q-3-grd** | **Rutin** | **Q-3-gsd** | **Quercetin** | **Cafft. A.** | **∑Polyphenols** |
| **Et25** | 5.3 ± 0.9 | 25 ± 1 | 21 ± 2 | 0.247 ± 0.002^H^ | 2.1 ± 0.5 | 49 ± 6 | 5.1 ± 0.3 | 0.70 ± 0.04 ^L^ | 5.6 ± 0.5 | 0.090 ± 0.001^H^ | 0.581 ± 0.003^L^ | 115 |
| **Et50** | 5.0 ± 0.1 | 42 ± 1 | 30.0 ± 0.4 | 0.324 ± 0.007^H^ | 16 ± 2^H^ | 134 ± 7^H^ | 14 ± 2^H^ | 2.3 ± 0.3 | 20 ± 3^H^ | 0.524 ± 0.001^H^ | 0.80 ± 0.02^L^ | 265^H^ |
| **Et75** | 4.0 ± 0.6 | 49 ± 2 | 34 ± 1 | 0.302 ± 0.001^H^ | 15.5 ± 0.4^H^ | 77 ± 2.3 | 12 ± 2.4 | 1.3 ± 0.35 | 14 ± 4.4 | 1.34 ± 0.015^H^ | 0.5 ± 0.02^L^ | 209 |
| **Et100** | 3.0 ± 0.1 | 62.5 ± 1.4 | 40 ± 7^H^ | 0.368 ± 0.004^H^ | 18 ± 1.3^H^ | 77.0 ± 0.8 | 12 ± 3 | 1.7 ± 0.1 | 16 ± 3 | 0.90 ± 0.03^H^ | 0.40 ± 0.01^L^ | 233 |
| **Lc25** | 10 ± 2^H^ | 39 ± 8 | 22 ± 1 | N/D^a^ | 4.0 ± 0.6 | 66 ± 1 | 10 ± 2 | 0.40 ± 0.02 ^L^ | 8.5 ± 0.4 | 1.99 ± 0.09^H^ | N/D | 161 |
| **Lc50** | 6.8 ± 0.6 ^H^ | 59 ± 7 | 24 ± 2 | 0.255 ± 0.009^H^ | 14 ± 2^H^ | 93 ± 9 | 22 ± 4^H^ | 0.50 ± 0.07 ^L^ | 26 ± 5^H^ | 5.4 ± 0.9^H^ | N/D | 252^H^ |
| **Lc75** | 3.0 ± 0.1 | 59 ± 2 | 21 ± 3 | 0.249 ± 0.001^H^ | 15 ± 1.0^H^ | 35 ± 2^L^ | 15 ± 2^H^ | 1.34 ± 0.01 | 17.9 ± 0.8 | 0.99 ± 0.06^H^ | N/D^a^ | 168 |
| **Lc100** | 2.5 ± 0.1 | 82.6 ± 0.3^H^ | 24 ± 9 | 0.262 ± 0.003^H^ | 18 ± 1^H^ | 37 ± 1^L^ | 16 ± 4^H^ | 1.50 ± 0.08 | 17 ± 1^H^ | 0.7 ± 0.2^H^ | N/D^a^ | 200 |
| **Pg25** | 8.8 ± 0.3^H^ | 34 ± 1 | 26 ± 1 | 0.247 ± 0.002^H^ | 2.0 ± 0.3 | 58 ± 9 | 6 ± 1 | 0.52 ± 0.08 ^L^ | 6 ± 1 | 0.11 ± 0.02^H^ | 0.8 ± 0.1^L^ | 142 |
| **Pg50** | 10 ± 2^H^ | 31 ± 4 | 25 ± 3 | 0.255 ± 0.008^H^ | 4.0 ± 0.2 | 58 ± 1.8 | 8 ± 1.9 | 0.80 ± 0.01 ^L^ | 6.7 ± 0.4 | 0.35 ± 0.072^H^ | 1.1 ± 0.44^L^ | 144 |
| **Pg75** | 11 ± 2^H^ | 40.5 ± 9 | 30 ± 3 | 0.268 ± 0.002^H^ | 8 ± 1^H^ | 85 ± 12 | 11.2 ± 0.6 | 0.63 ± 0.08 ^L^ | 11.5 ± 0.9 | 1.2 ± 0.5^H^ | 1.4 ± 0.7 | 201 |
| **Pg100** | 5.7 ± 0.6 | 40.3 ± 4.8 | 31 ± 2.1 | 0.275 ± 0.007^H^ | 12.3 ± 0.8^H^ | 65 ± 0.3 | 8 ± 1.2 | 0.76 ± 0.07 ^L^ | 8.3 ± 0.8 | 1.2 ± 0.12^H^ | 0.8 ± 0.1^L^ | 174 |
| **W100** | 3.7 ± 0.1 | 30 ± 2 | 22.0 ± 0.2 | N/D | 2.2 ± 0.3 | 84 ± 3 | 5.5 ± 0.6 | 1.8 ± 0.2 | 5.7 ± 0.7 | N/D^a^ | 2.07 ± 0.04 | 157 |
| **LSD Test** | *p*<0.001 | *p*>0.05 | *p*>0.0.5 | *p*<0.001 | *p*<0.001 | *p*<0.004 | *p*<0.001 | *p*<0.001 | *p*<0.003 | *p*<0.001 | *p*>0.050 | *p*<0.025 |

^a^N/D: No detected ^H^Upper significant value ^L^Lower significant value. Gallic A: Gallic acid; Epi.: Epicatechin; Epigallo. g.: Epigallocatechin gallate; Epica. g.: Epicatechin gallate; Q-3-grd: Quercetin-3-glucuronide; Q-3-gsd: Quercetin-3-glucoside; Cafft. A.:Caftaric acid; ΣProcyan.: Total procyanidins (B1+B2+C1).

**Supplementary Table 4.** Stability of the polyphenolic profile present in grape marc extract to changes in storage parameters: temperature, light, and oxidative reactivity, for 62 days of analysis.

|  |  | **Concentration (mg·L-1)** | | | | | | | | | | | |
| --- | --- | --- | --- | --- | --- | --- | --- | --- | --- | --- | --- | --- | --- |
| **Factor** | **Value** | **Catec.** | **Epic.** | **Q-3-grd** | **Rutin** | **Q-3-gsd** | **Querc.** | **Gallic A.** | **Caft. A.** | **ΣProcyan.**  **(B1+B2+C1)** | **Epigallo. g.** | **Epica. g.** | **Σ Total** |
| **Day** | 0 | 65.5 | 31.3 | 20.2 | 3.06 | 26.5 | 0.63 | 1.91 | 0.378 | 107 | 0.006 | 14.6 | 270 |
|  | 2 | 64.8 | 30.1 | 20.6 | 2.26^L^ | 26.6 | 1.74^H^ | 4.15 | 0.439 | 115 | 0.016 | 14.5 | 280 |
|  | 4 | 60.7 | 29.8 | 20.8 | 1.80^L^ | 25.9 | 2.38^H^ | 4.25 | 0.513 | 100 | 0.018 | 12.7 | 260 |
|  | 8 | 66.6 | 29.9 | 20.3 | 1.39^L^ | 23.9 | 3.18^H^ | 4.12 | 0.421 | 89.8^L^ | 0.016 | 13.9 | 254 |
|  | 20 | 55.4 | 24.0 | 17.9 | 0.838^L^ | 18.6^L^ | 4.09^H^ | 5.52^H^ | 0.254 | 66.3^L^ | N/D | 10.3^L^ | 204^L^ |
|  | 34 | 112^H^ | 48.4^H^ | 15.0^L^ | 0.693^L^ | 11.5^L^ | 5.84^H^ | 7.09^H^ | 0.237 | 54.7^L^ | N/D | 13.6 | 270 |
|  | 62 | 60.3 | 23.8 | 16.1 | 0.442^L^ | 12.4^L^ | 5.12^H^ | 7.30^H^ | 0.202^L^ | 50.1^L^ | N/D | 8.35^L^ | 186^L^ |
| ***p*-value** | | <0.001 | <0.001 | <0.001 | <0.001 | <0.001 | <0.001 | <0.001 | <0.001 | <0.001 | <0.001 | <0.001 | <0.001 |
| **Light** | No | 69.4 | 31.2 | 18.7 | 1.47 | 20.8 | 3.09 | 4.79 | 0.348 | 84.0 | 0.008 | 12.6 | 247 |
|  | Yes | 69.2 | 30.9 | 18.7 | 1.53 | 20.7 | 2.97 | 5.02 | 0.351 | 82.7 | 0.008 | 12.5 | 245 |
| ***p*-value** | | 0.885 | 0.656 | 0.864 | 0.207 | 0.858 | 0.513 | 0.239 | 0.804 | 0.309 | 0.685 | 0.497 | 0.633 |
| **N2** | No | 67.7 | 30.3 | 18.1 | 1.47 | 20.4 | 2.71 | 5.03 | 0.334 | 80.1 | 0.007 | 12.3 | 239 |
|  | Yes | 70.9^H^ | 31.8^H^ | 19.0 | 1.52 | 21.1 | 3.35^H^ | 4.79 | 0.365^H^ | 86.6^H^ | 0.009^H^ | 12.8^H^ | 253^H^ |
| ***p*-value** | | 0.025 | 0.006 | 0.124 | 0.262 | 0.070 | 0.001 | 0.223 | 0.011 | <0.001 | 0.037 | 0.024 | <0.001 |
| **T (ºC)** | -20 | 72.3^H^ | 33.3^H^ | 18.5 | 2.44^H^ | 22.3^H^ | 0.525^L^ | 3.37^L^ | 0.347 | 93.0^H^ | 0.005^L^ | 14.7^H^ | 261^H^ |
|  | 4 | 70.3^H^ | 31.5^H^ | 18.8 | 1.16^H^ | 20.4 | 3.35^L^ | 5.25^L^ | 0.360 | 83.2^H^ | 0.007^L^ | 12.0^H^ | 247^H^ |
|  | 20 | 65.3 | 28.3 | 18.9 | 0.896 | 19.6 | 5.20 | 6.10 | 0.340 | 73.9 | 0.012 | 11.0 | 230 |
| ***p*-value** | | <0.001 | <0.001 | 0.643 | <0.001 | <0.001 | <0.001 | <0.001 | 0.401 | <0.001 | <0.001 | <0.001 | <0.001 |

Catec.: Catechin; Epic.:Epicatechin; Q-3-grd: Quercetin-3-glucuronide; Q-3-gsd: Quercetin-3-glucoside; Querc.: Quercetin; Gallic A.: Gallic acid; Caft. A.:Caftaric acid; ΣProcyan.: Total procyanidins; Epigallo. g.: Epigallocatechin gallate; Epica. g.: Epicatechin gallate. ^H^Upper significant value; ^L^Lower significant value.

| **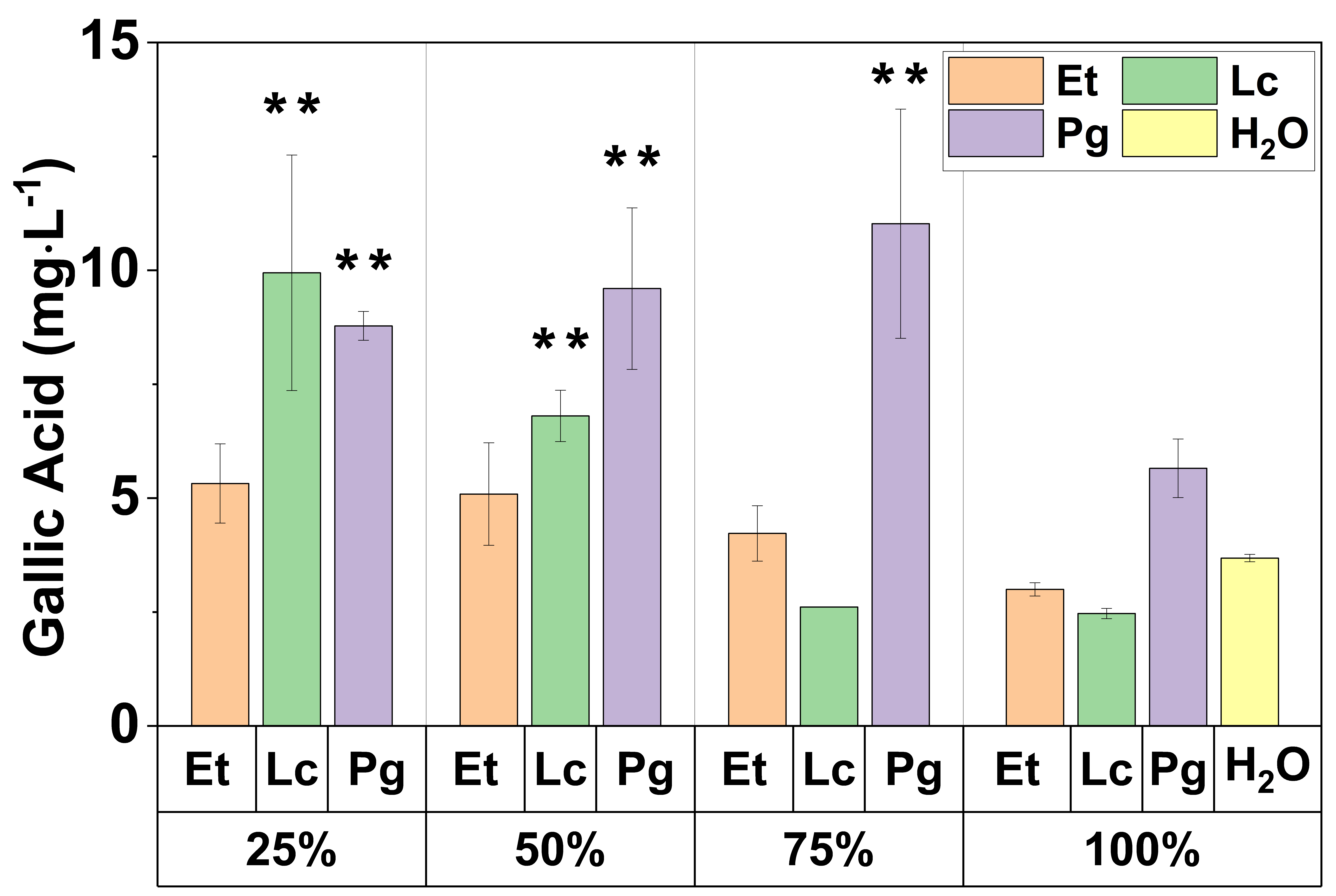** | **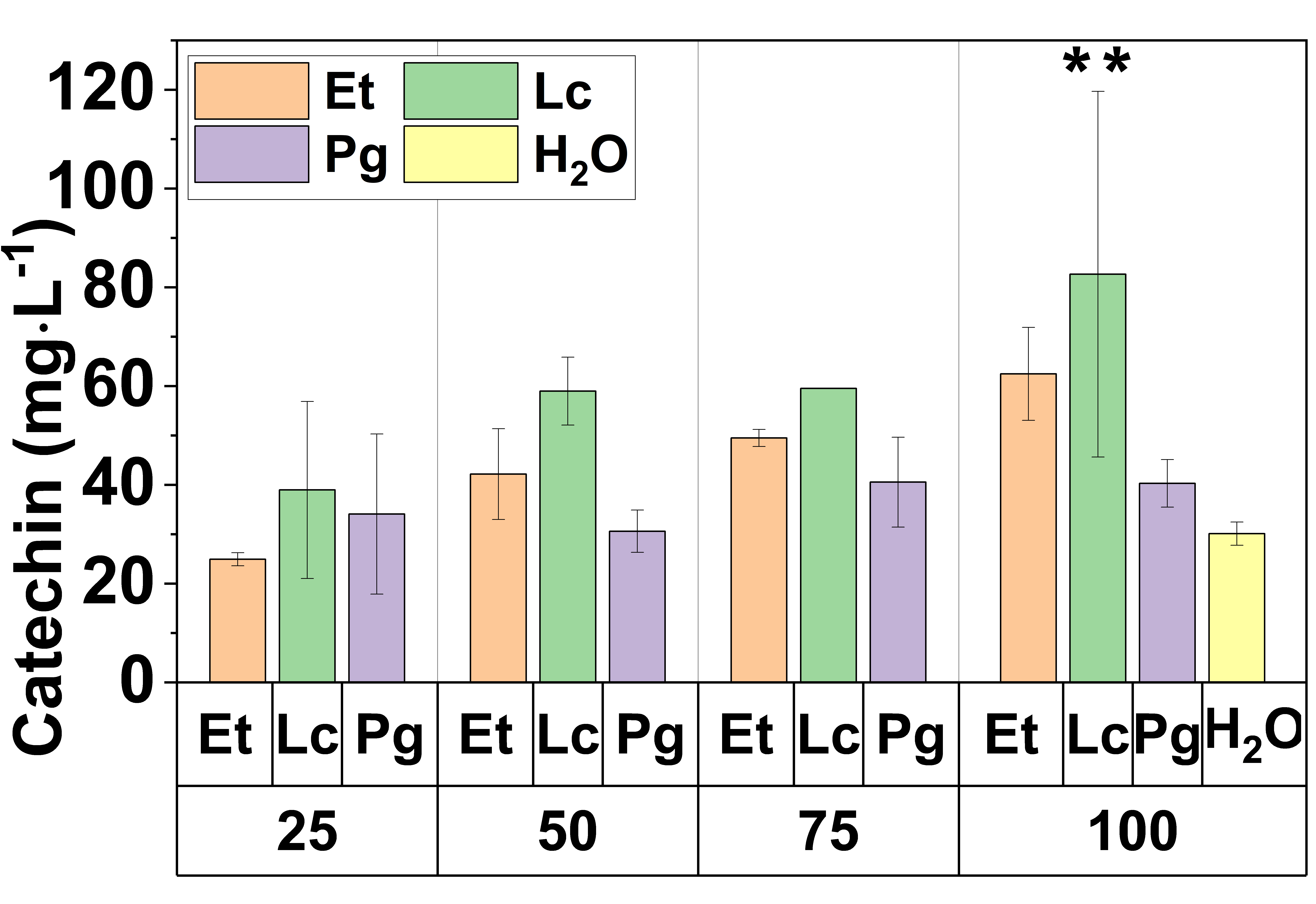** | **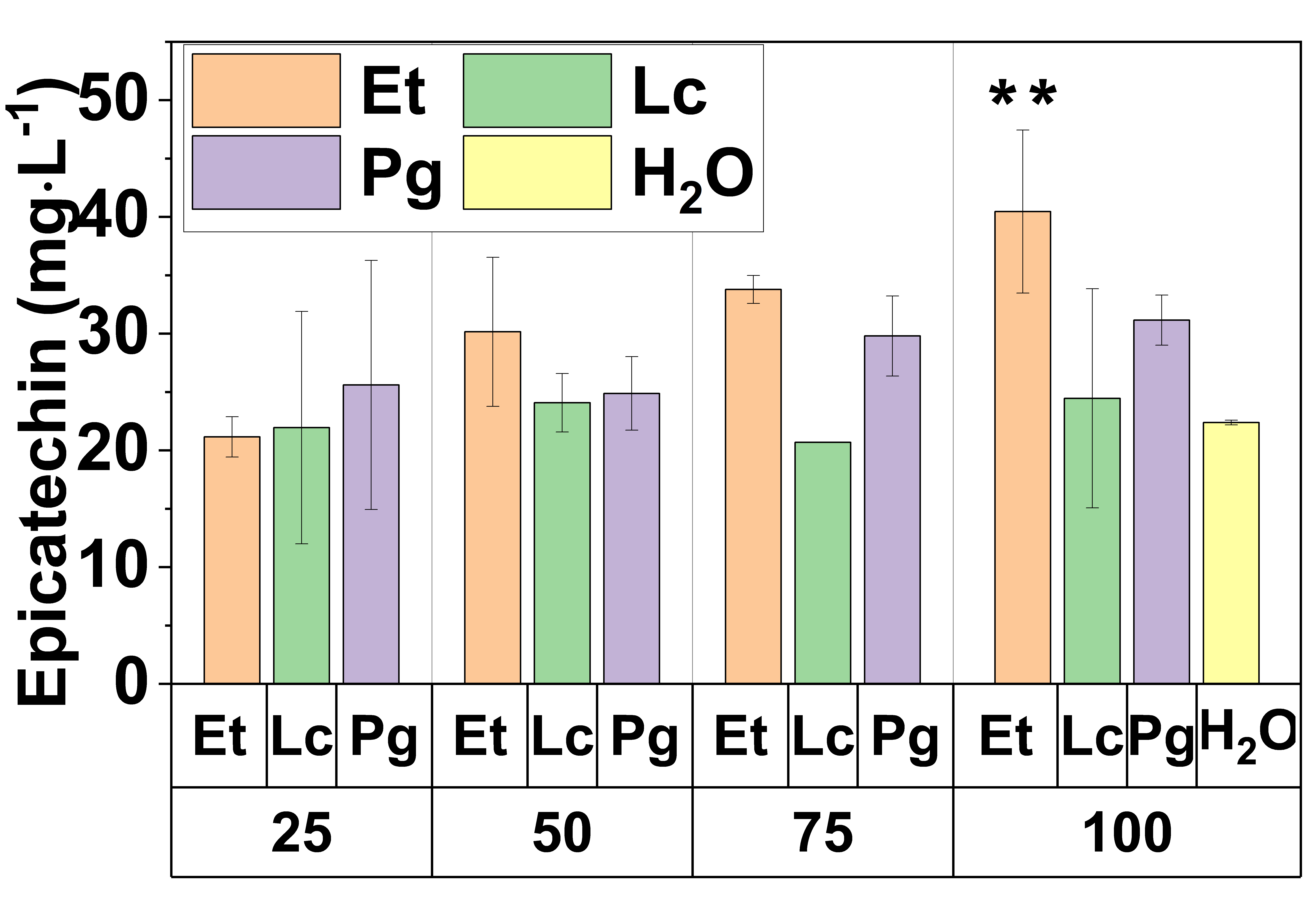** |
| --- | --- | --- |
| **(A)** | **(B)** | **(C)** |
| **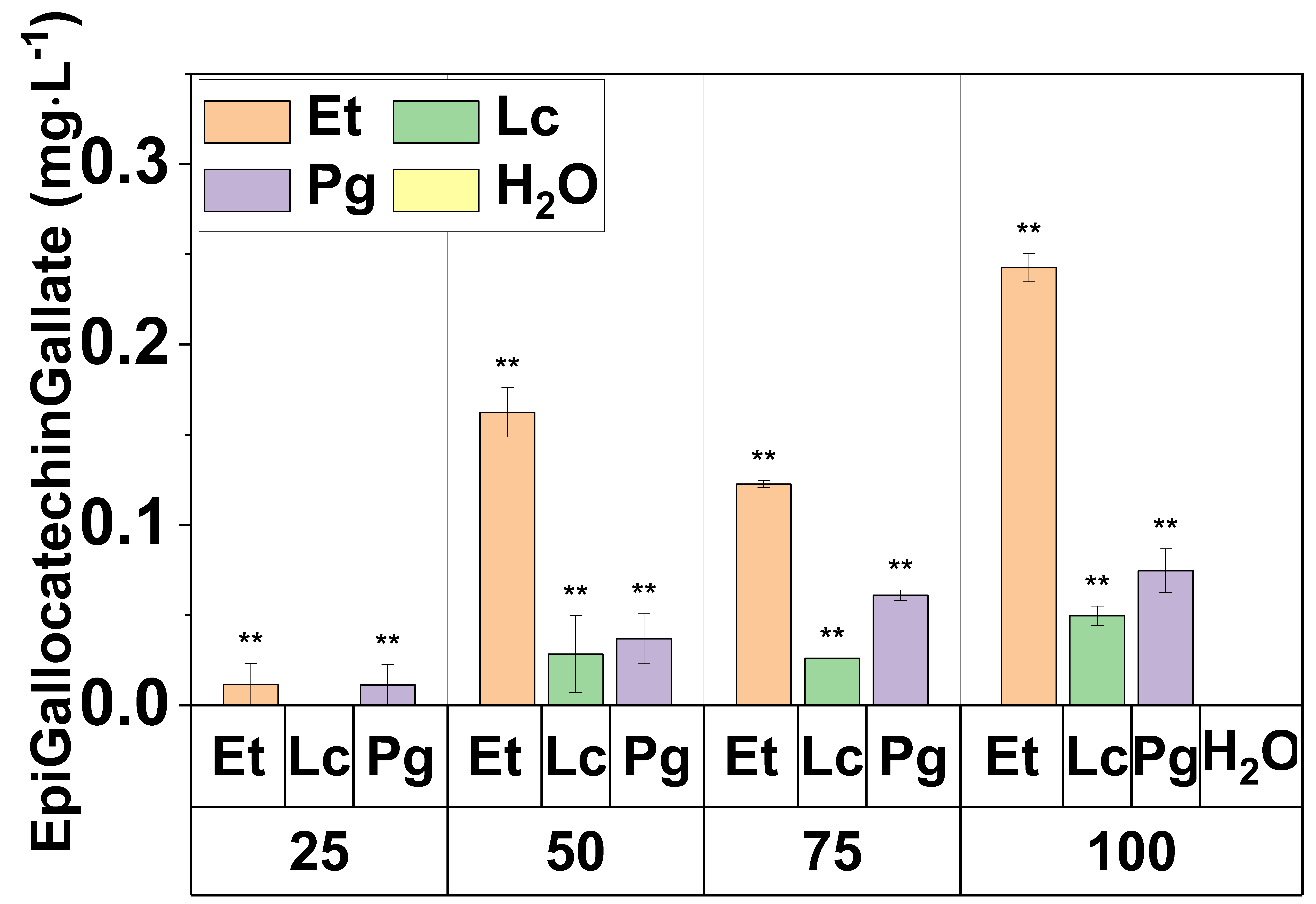** | **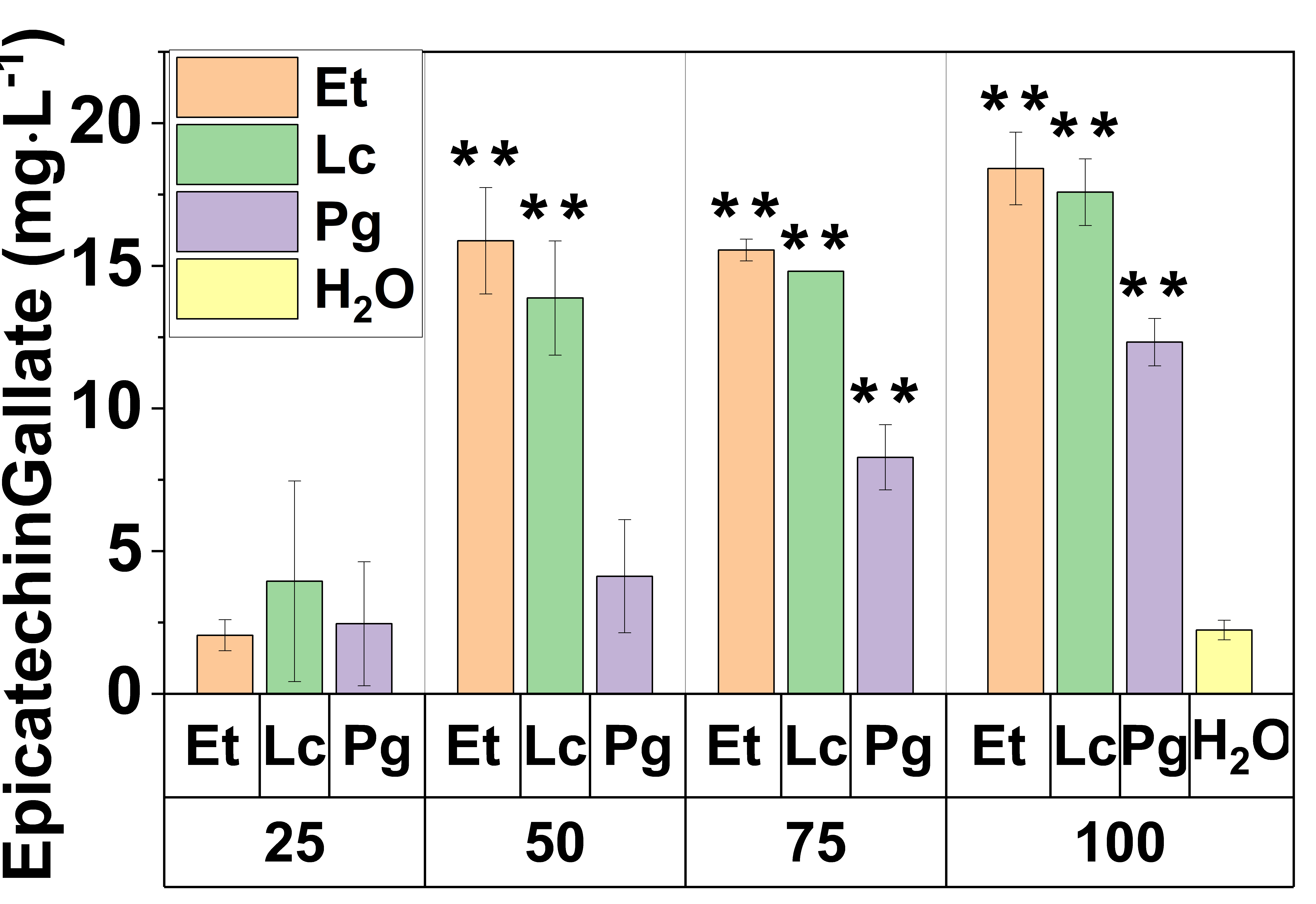** | **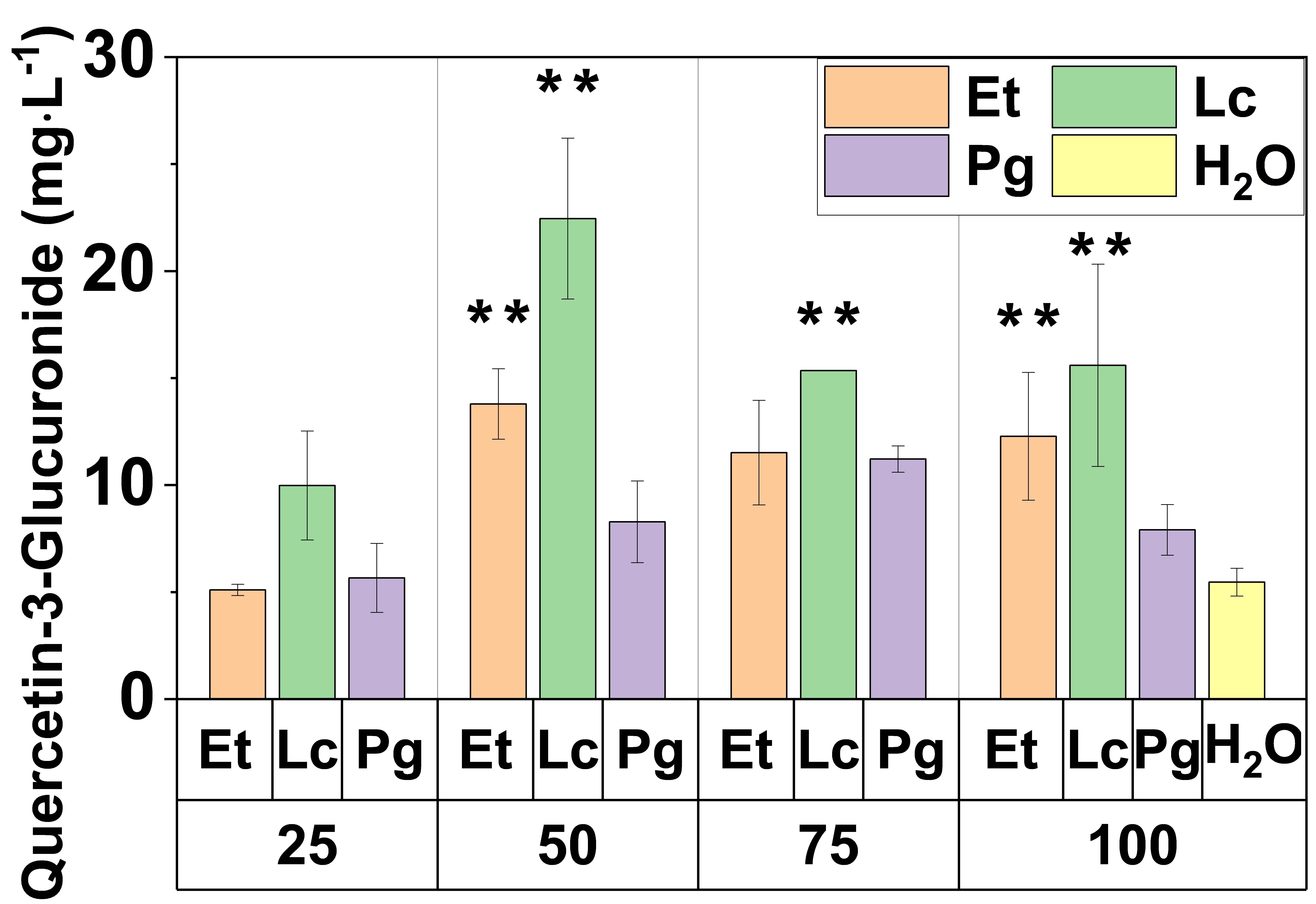** |
| **(D)** | **(E)** | **(F)** |
| **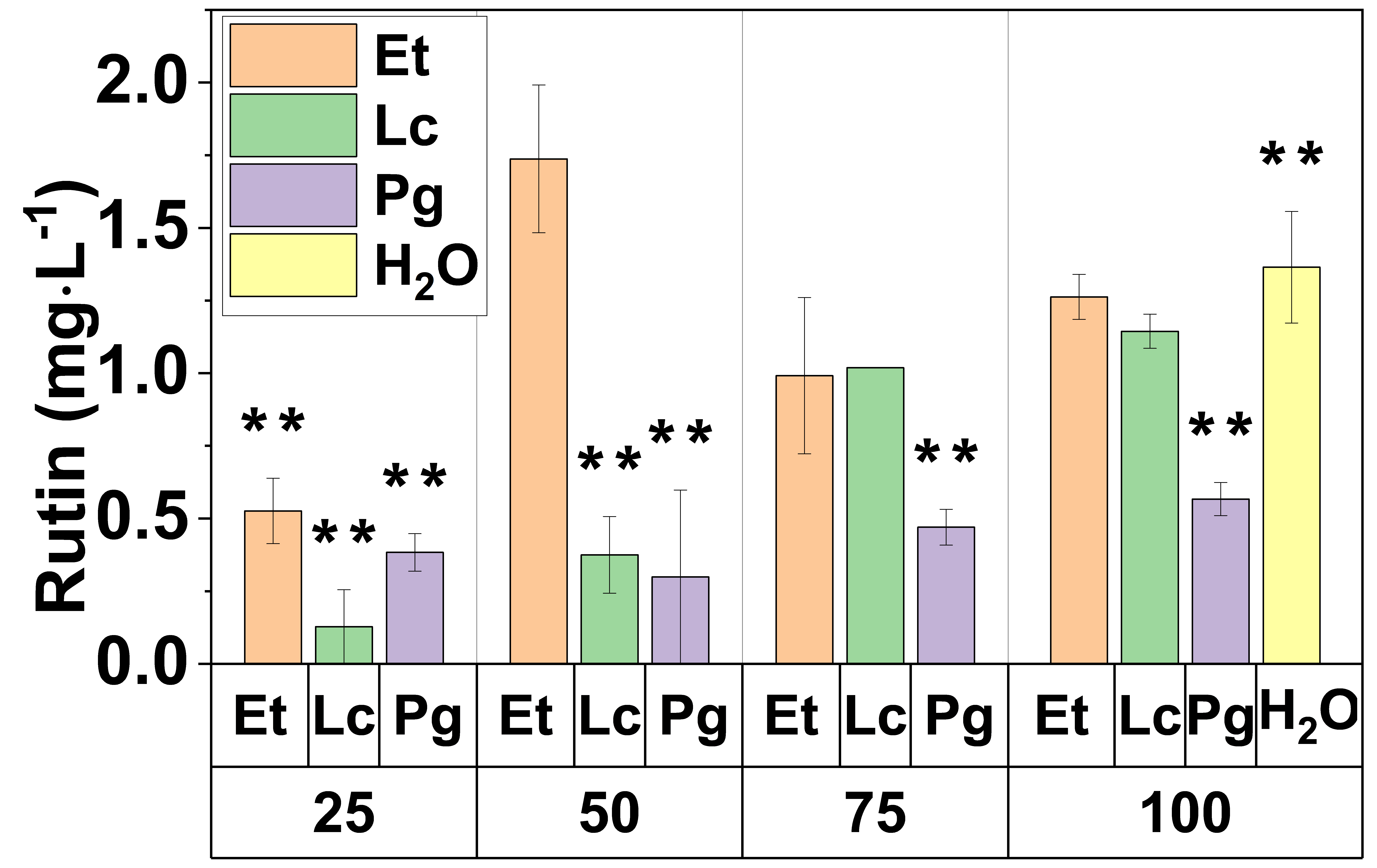** | **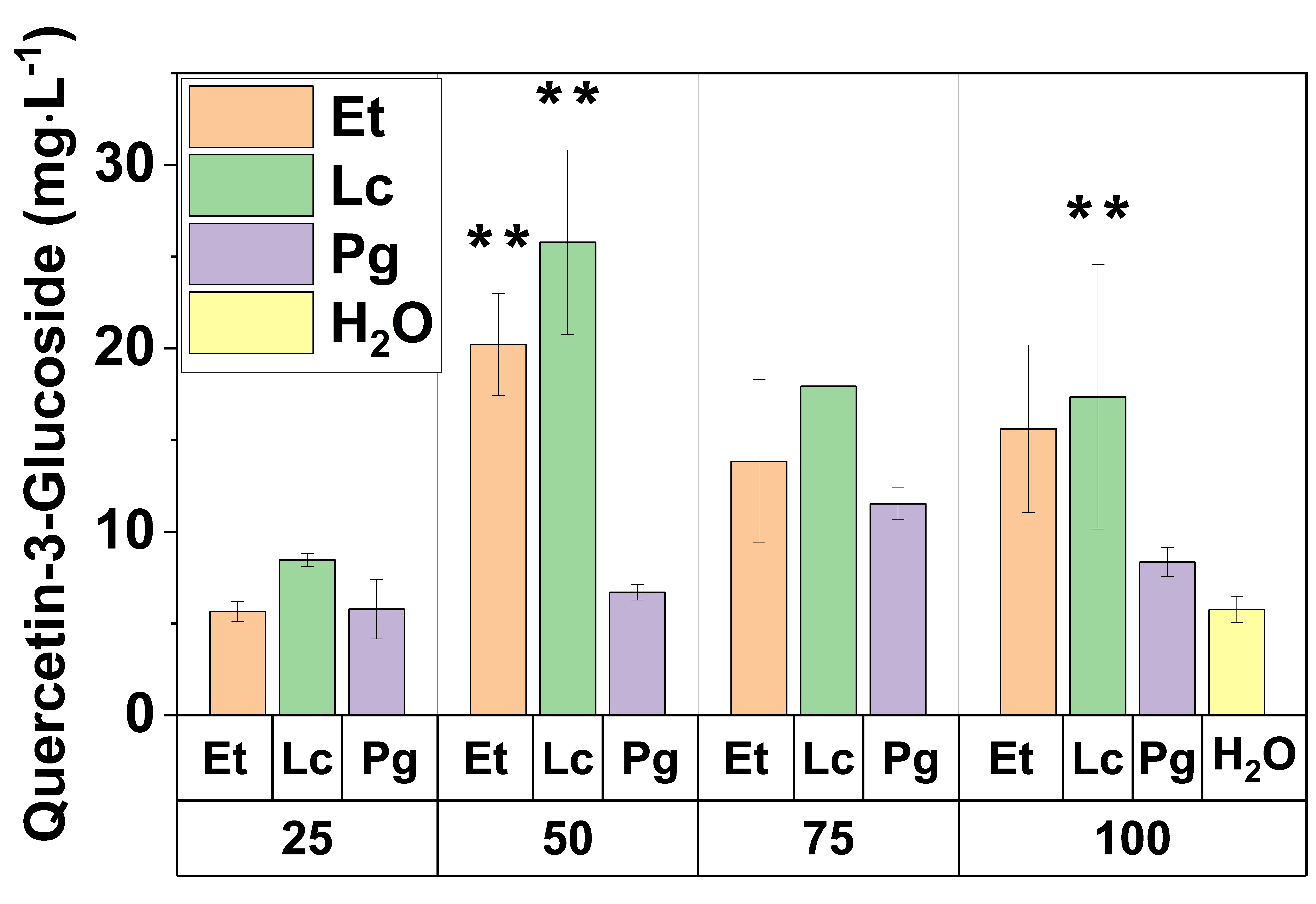** | **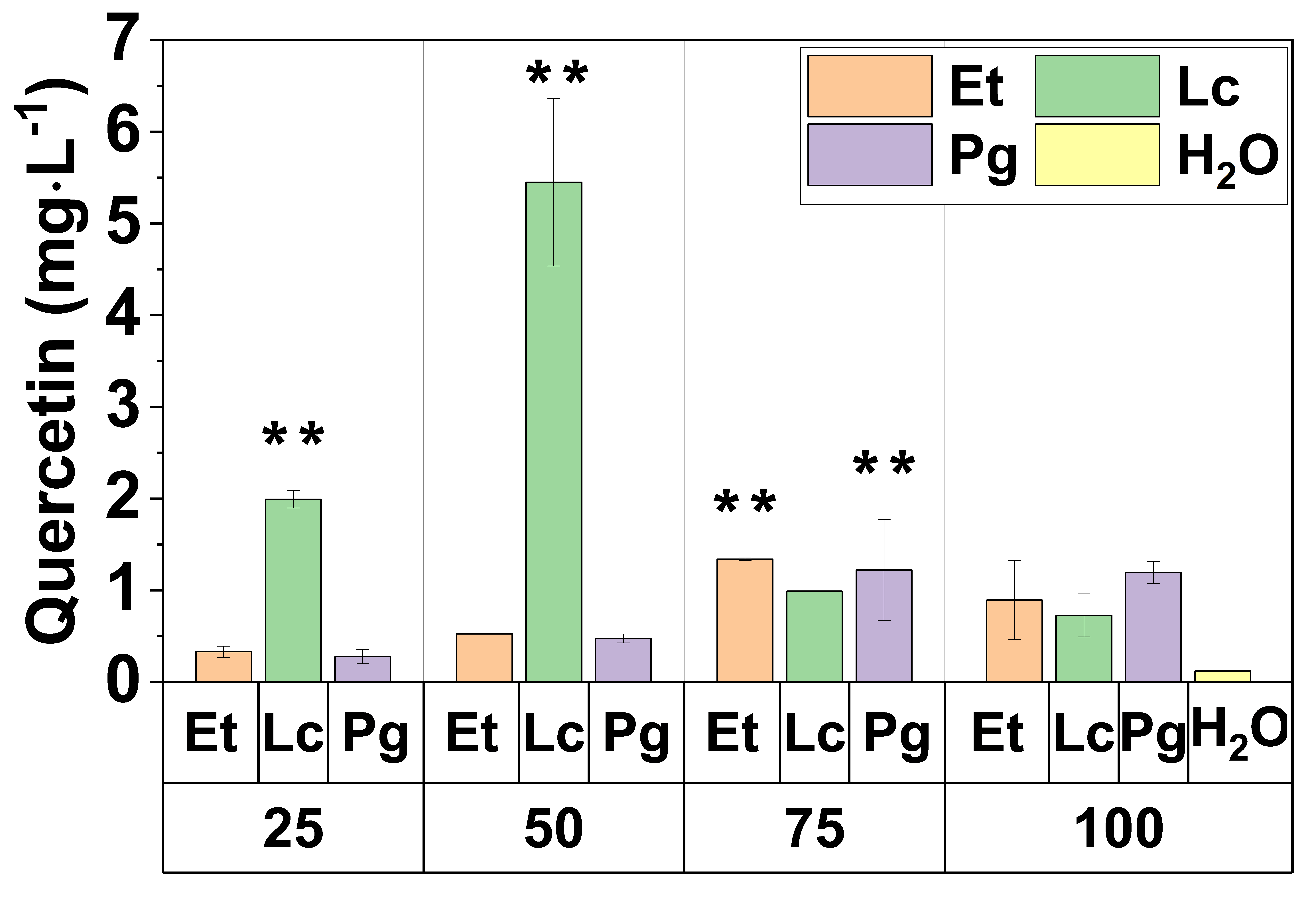** |
| **(G)** | **(H)** | **(I)** |
| **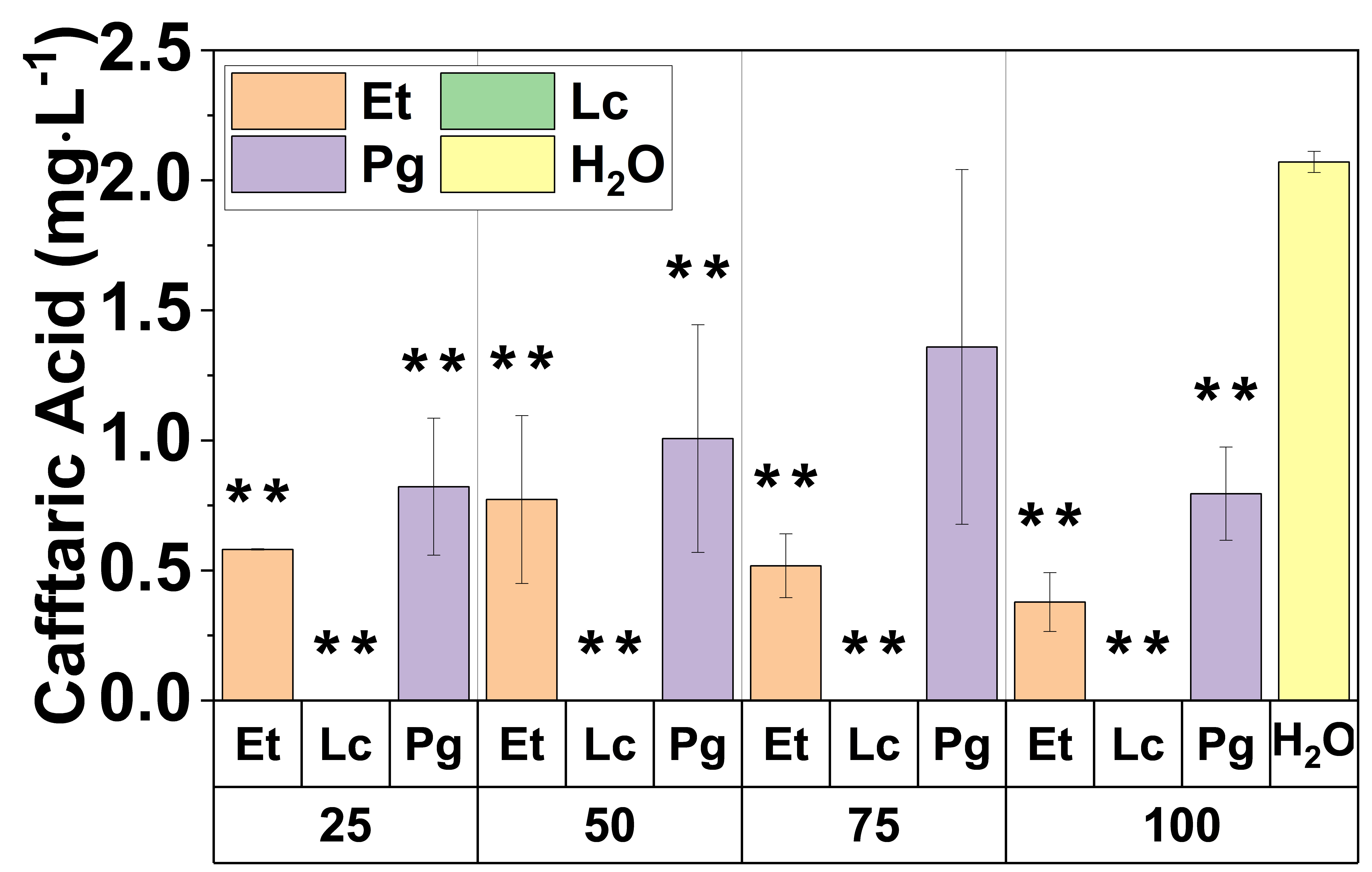** | **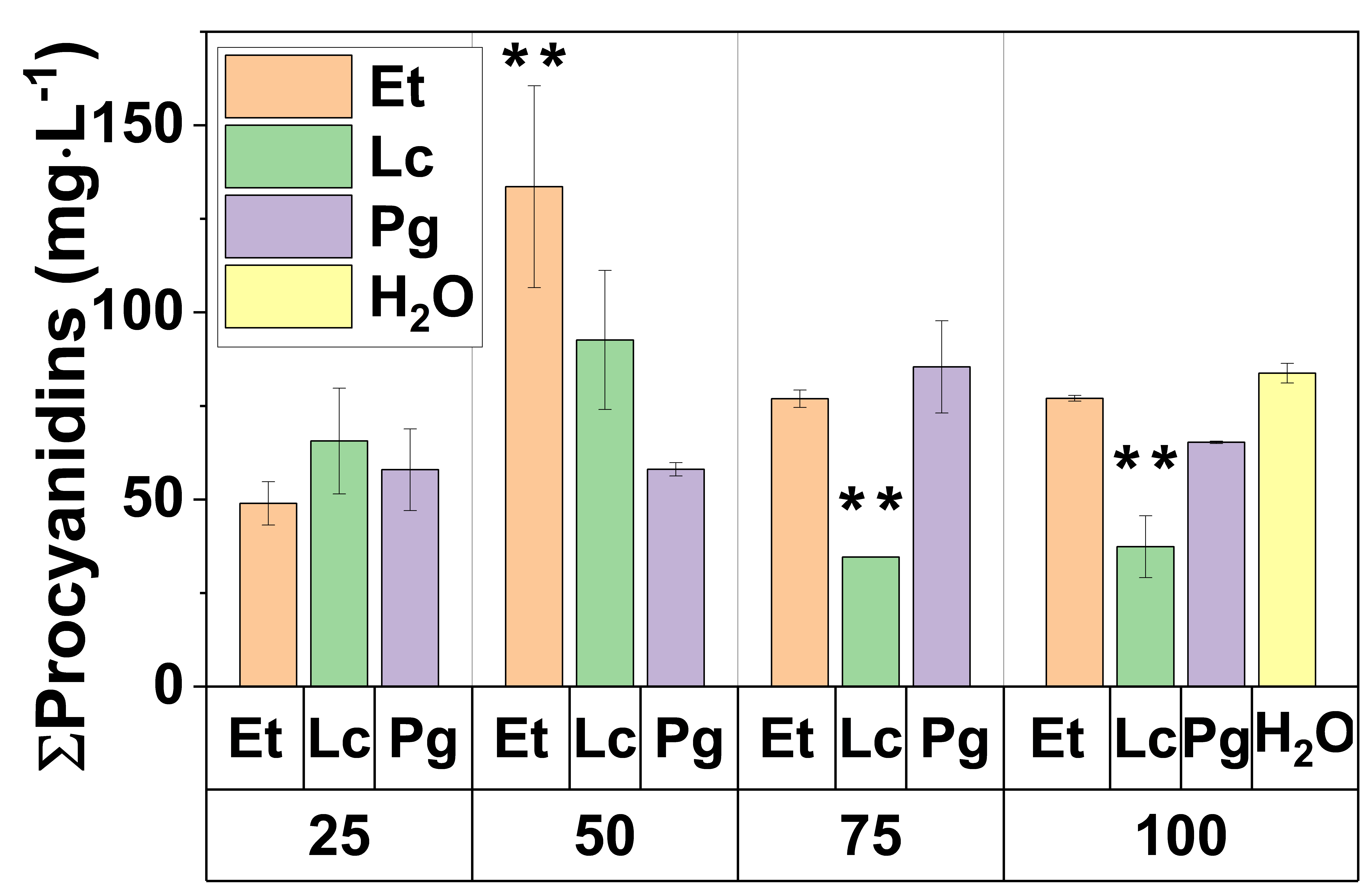** | **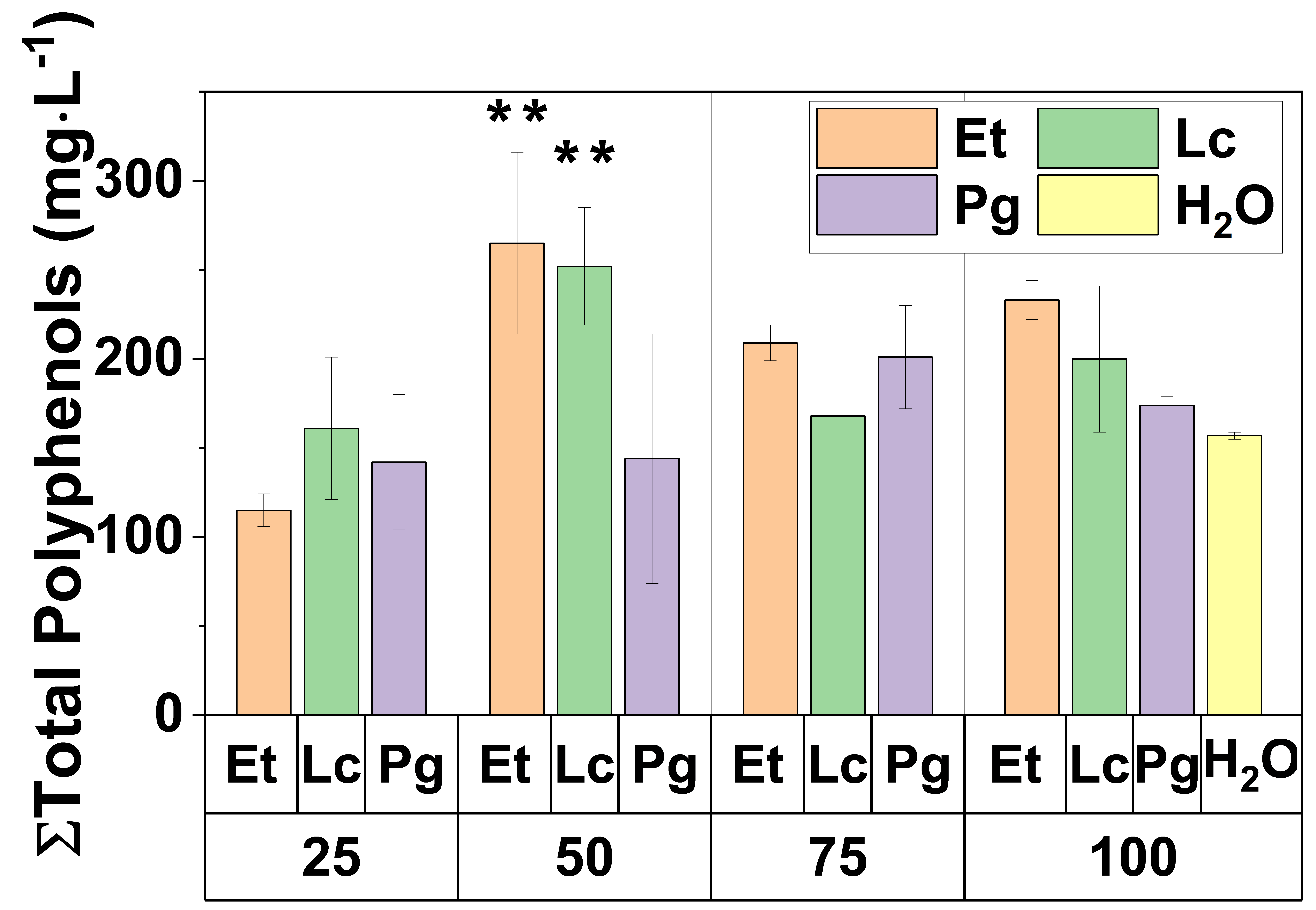** |
| **(J)** | **(K)** | **(L)** |

**Supplementary Figure 1.** Individual concentration of the main polyphenols contained in the white grape marc extracts to modifications in the proportions of the extractive solvents. H_2_O. Water; Et. Ethanol; Lc. Ethyl lactate; Pg. Propylene glycol. **(A)**. Gallic acid; **(B)**. Catechin; **(C)**. Epicatechin; **(D)**. Epigallocatechin gallate; **(E)**. Epicatechin gallate; **(F)**. Quercetin-3-glucuronide; **(G)**. Rutin; **(H)**. Quercetin-3-glucoside; **(I)**. Quercetin; **(J)**. Cafftaric Acid; **(K)**. Total procyanidins; **(L)**. Total polyphenols. ****** significant value

| 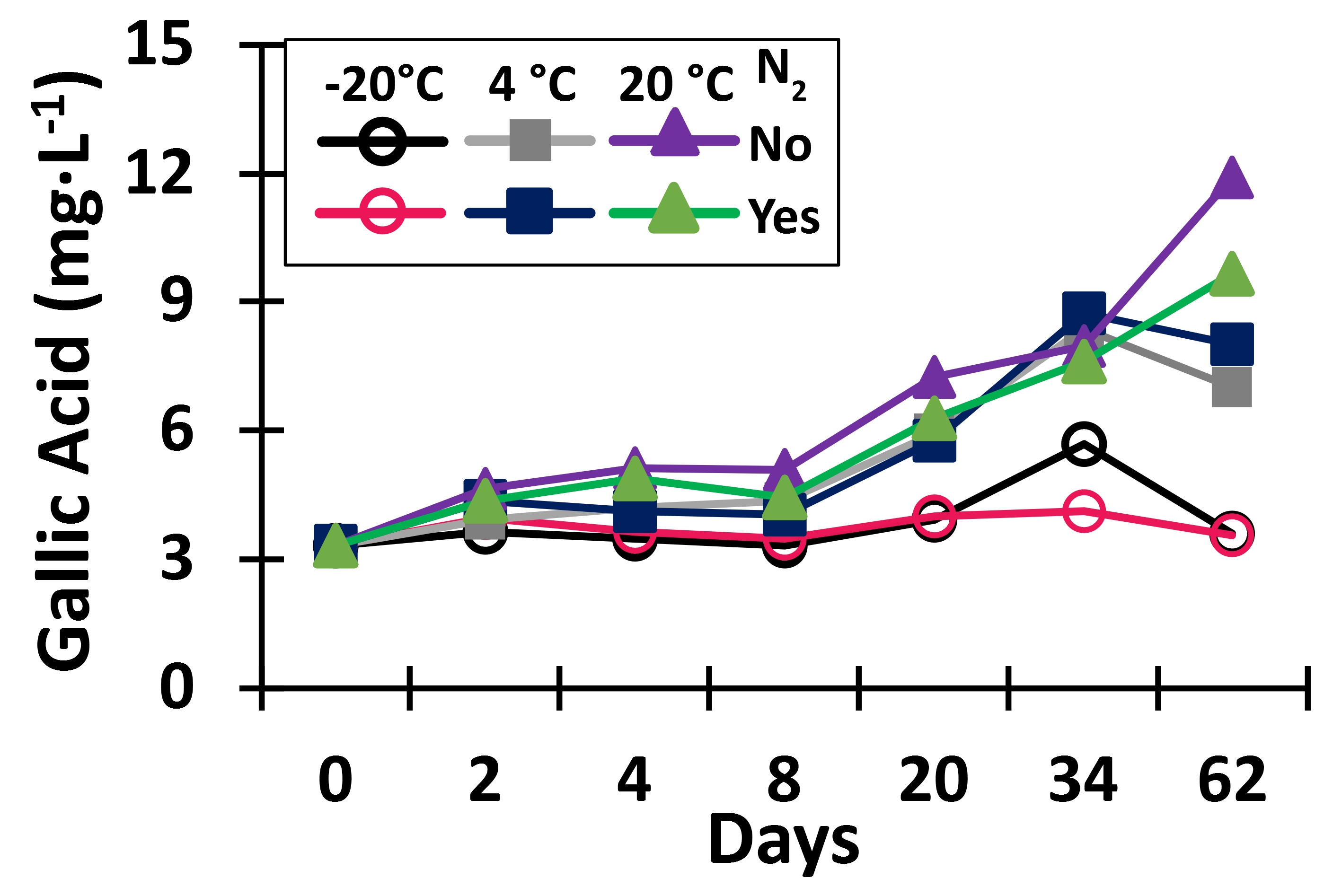 | 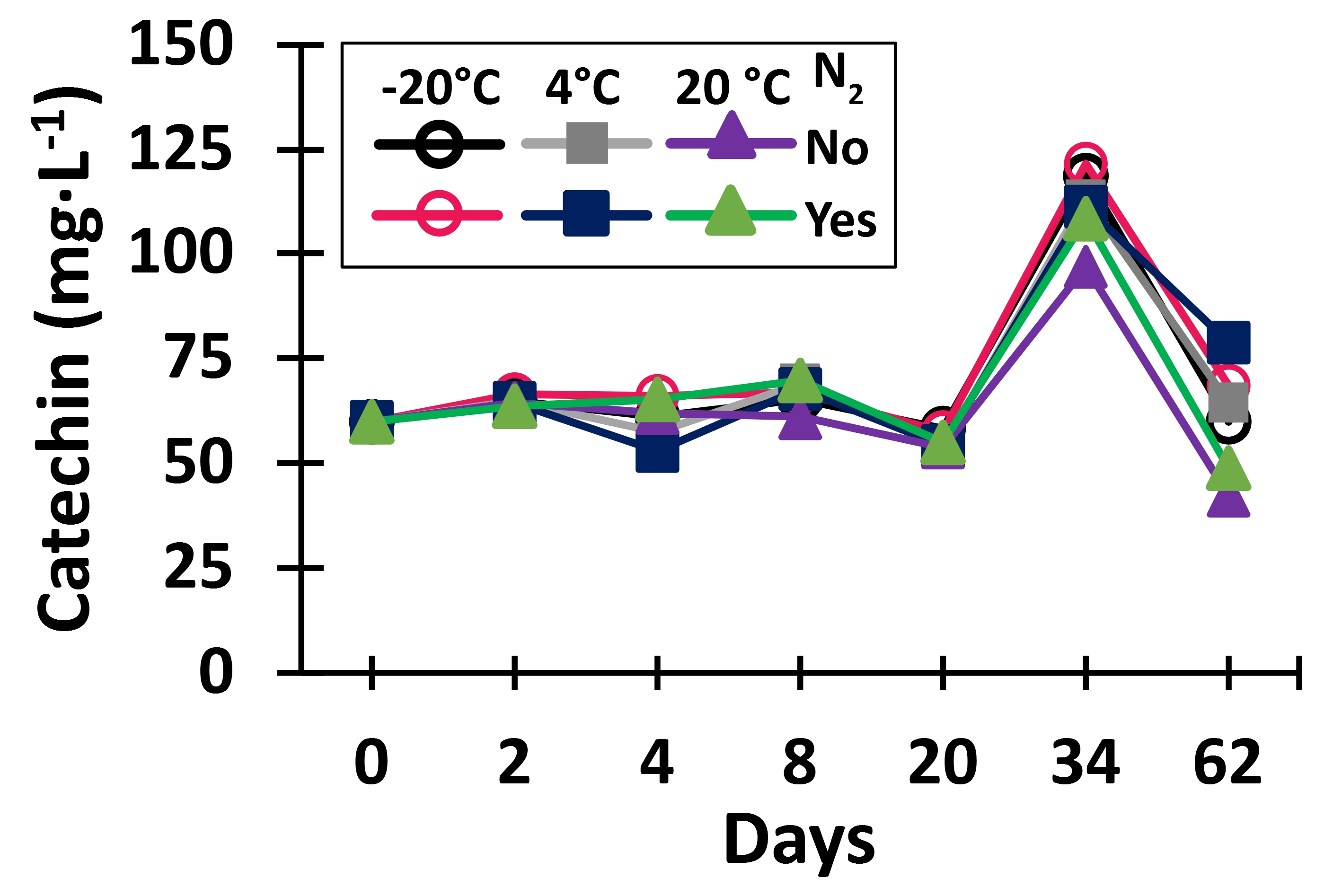 | 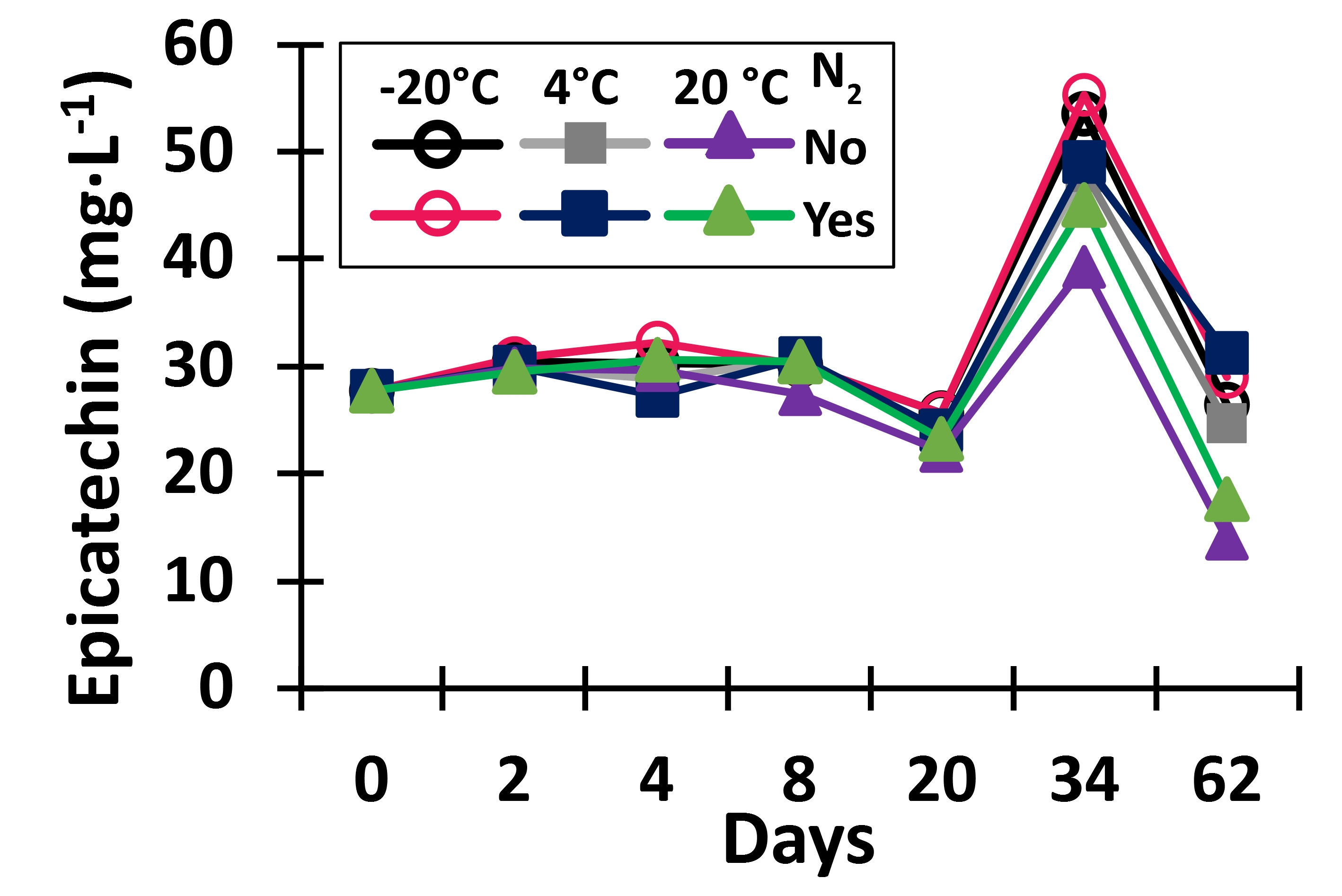 |
| --- | --- | --- |
| **(A)** | **(B)** | **(C)** |
| 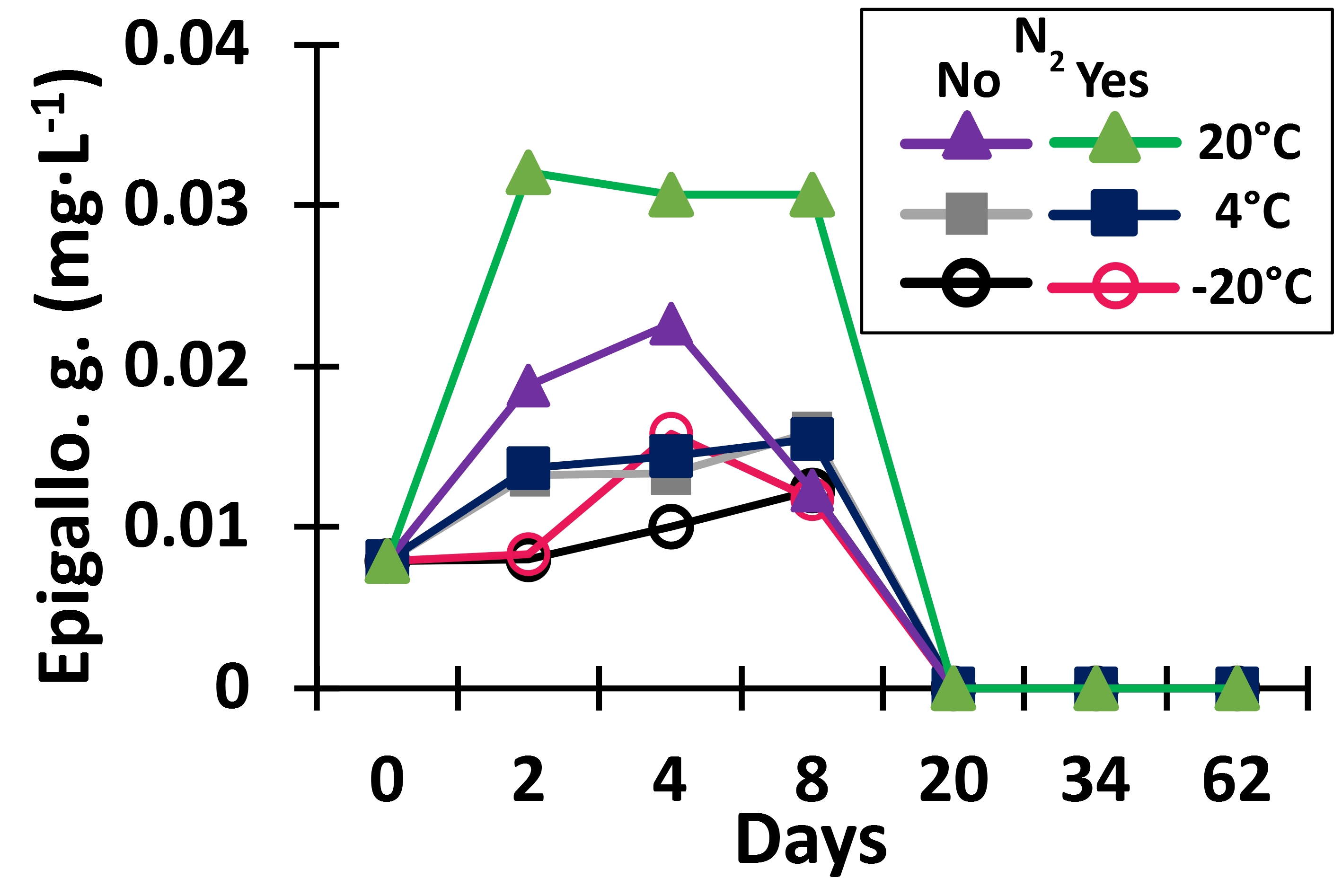 | 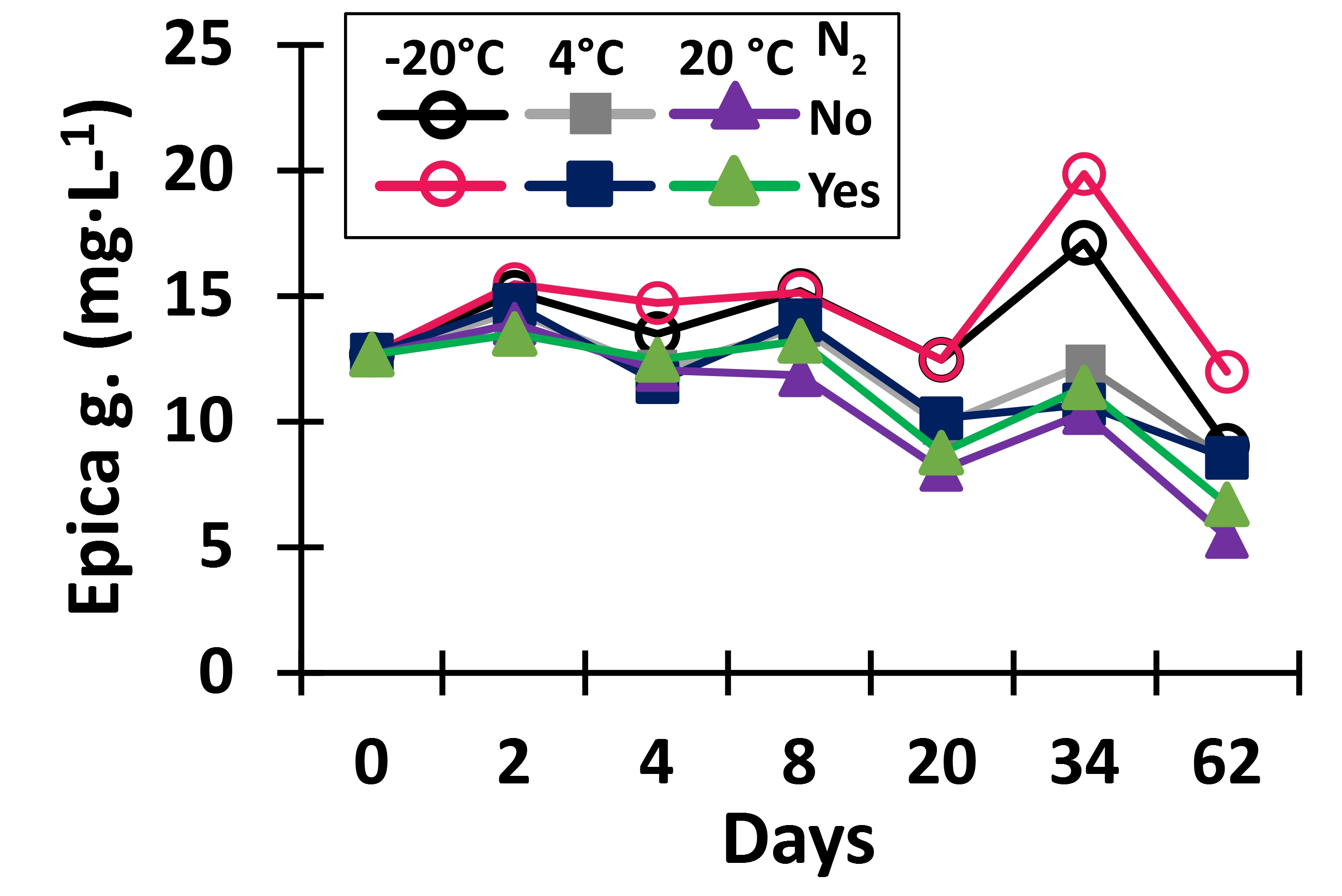 | 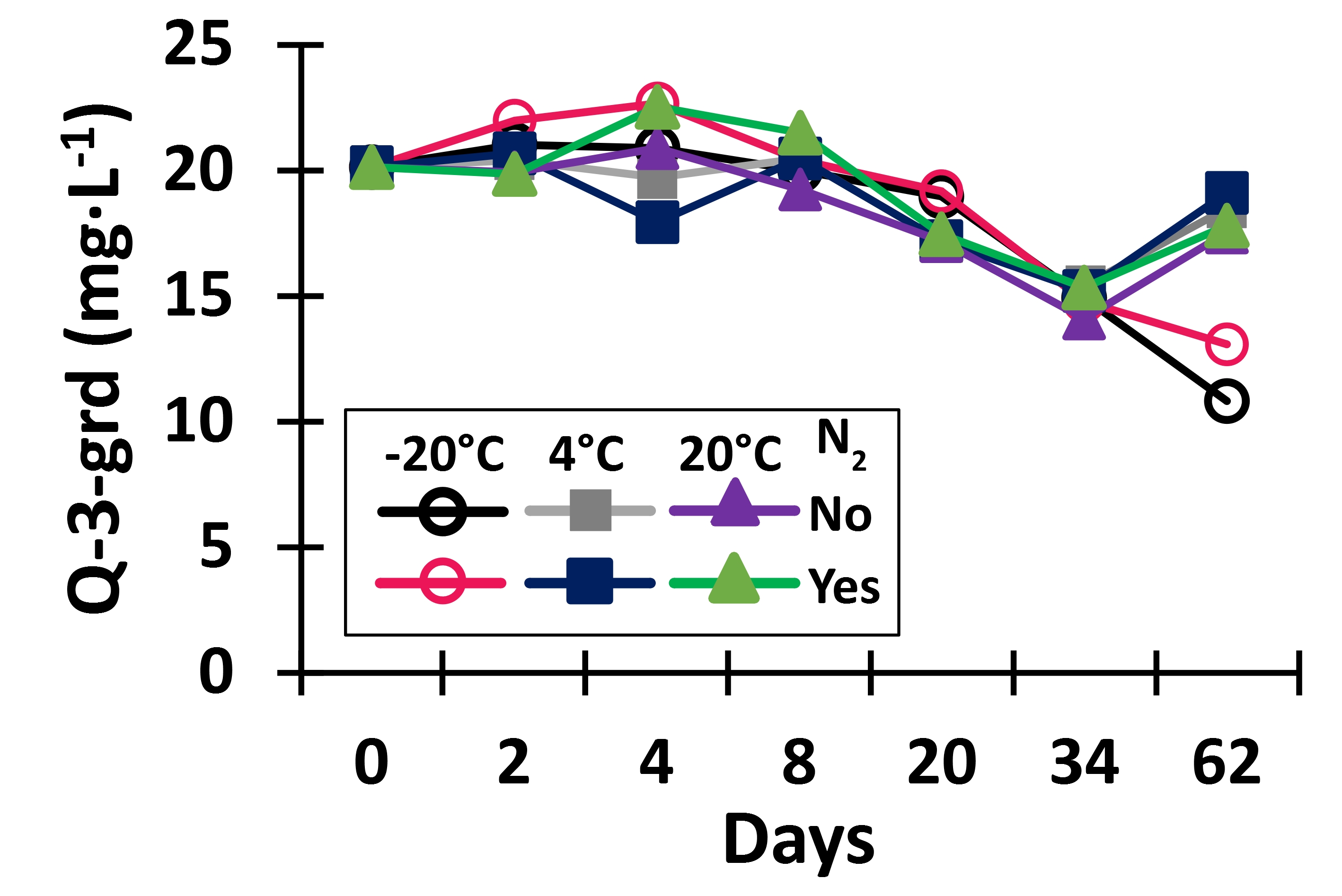 |
| **(D)** | **(E)** | **(F)** |
| 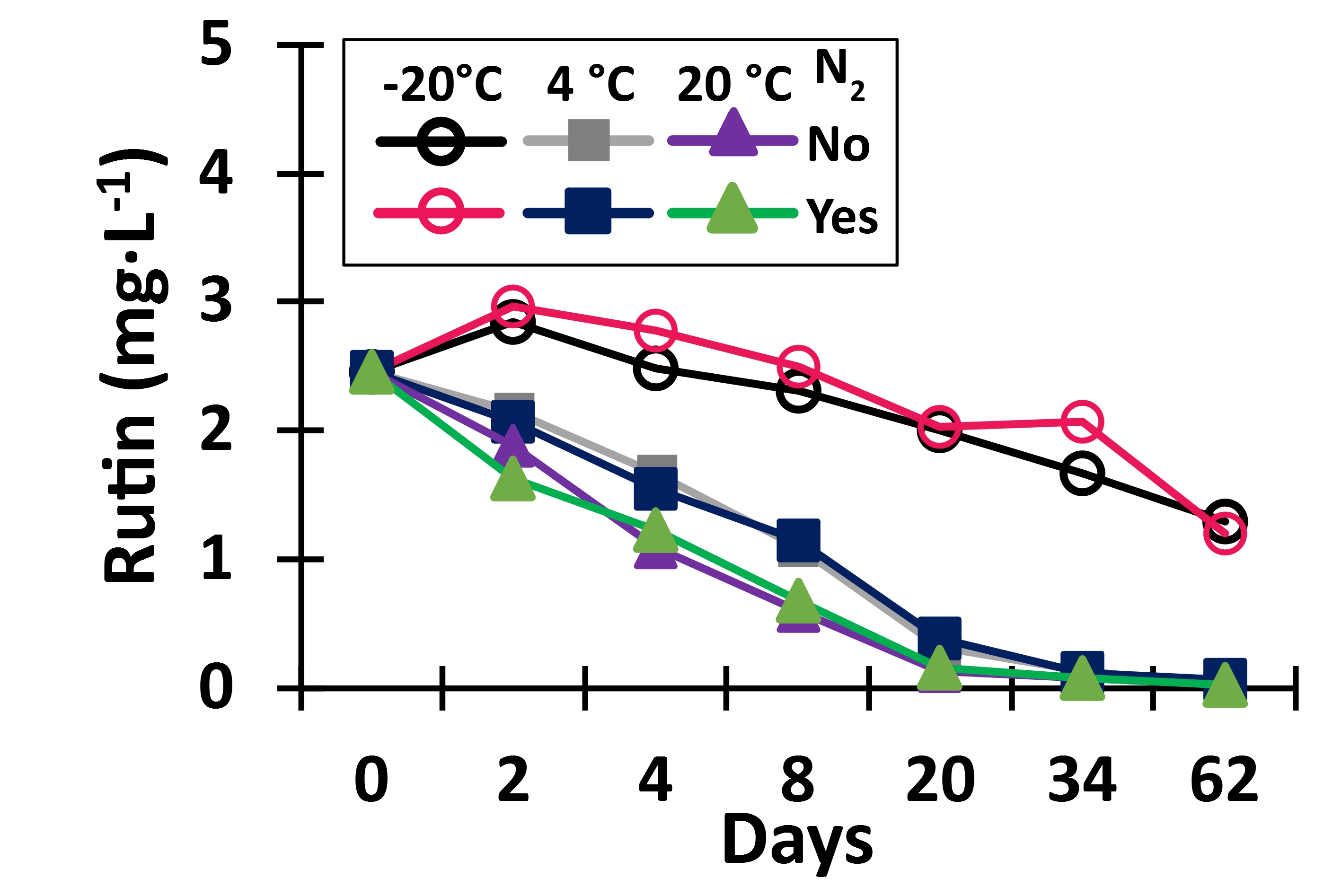 | 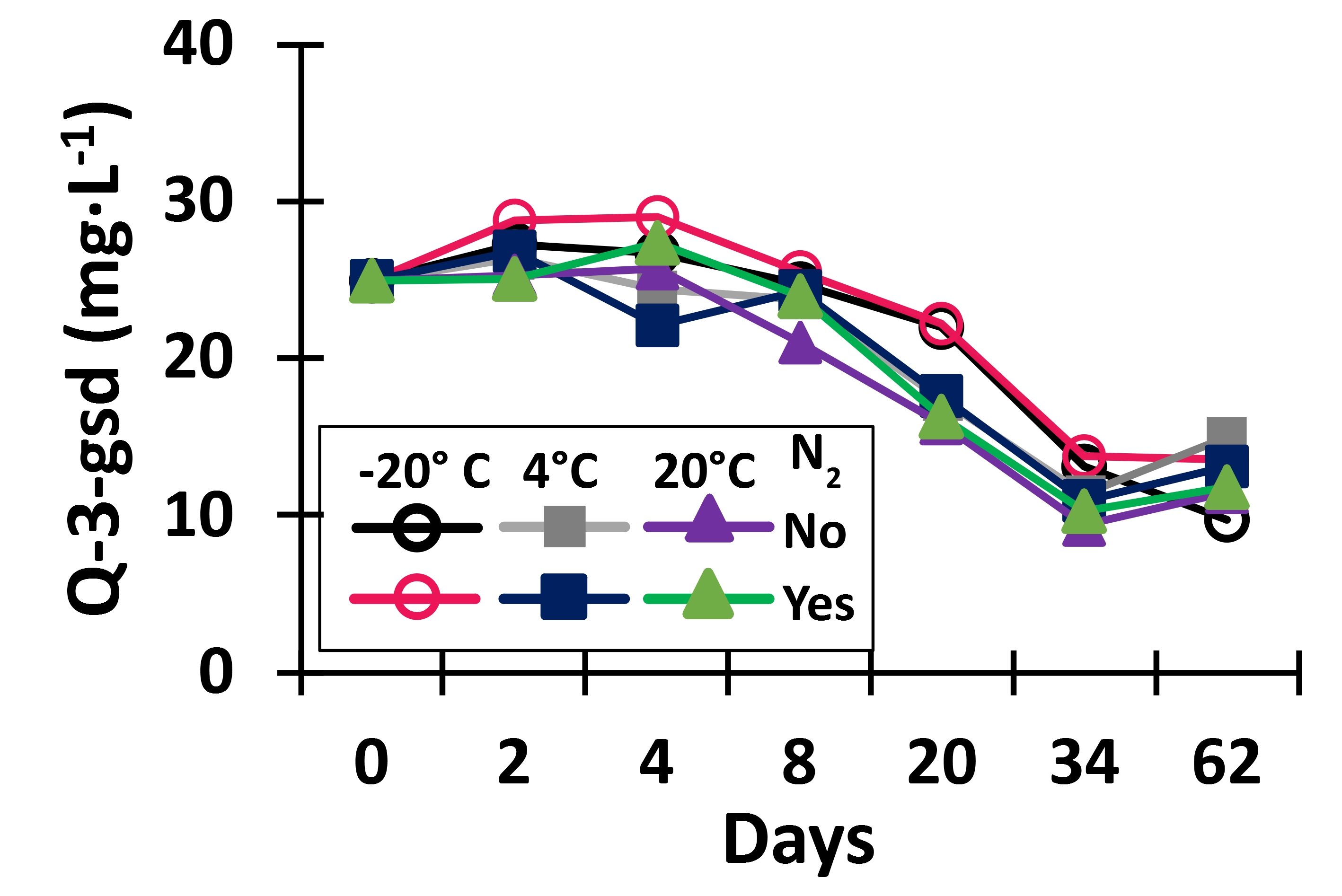 | 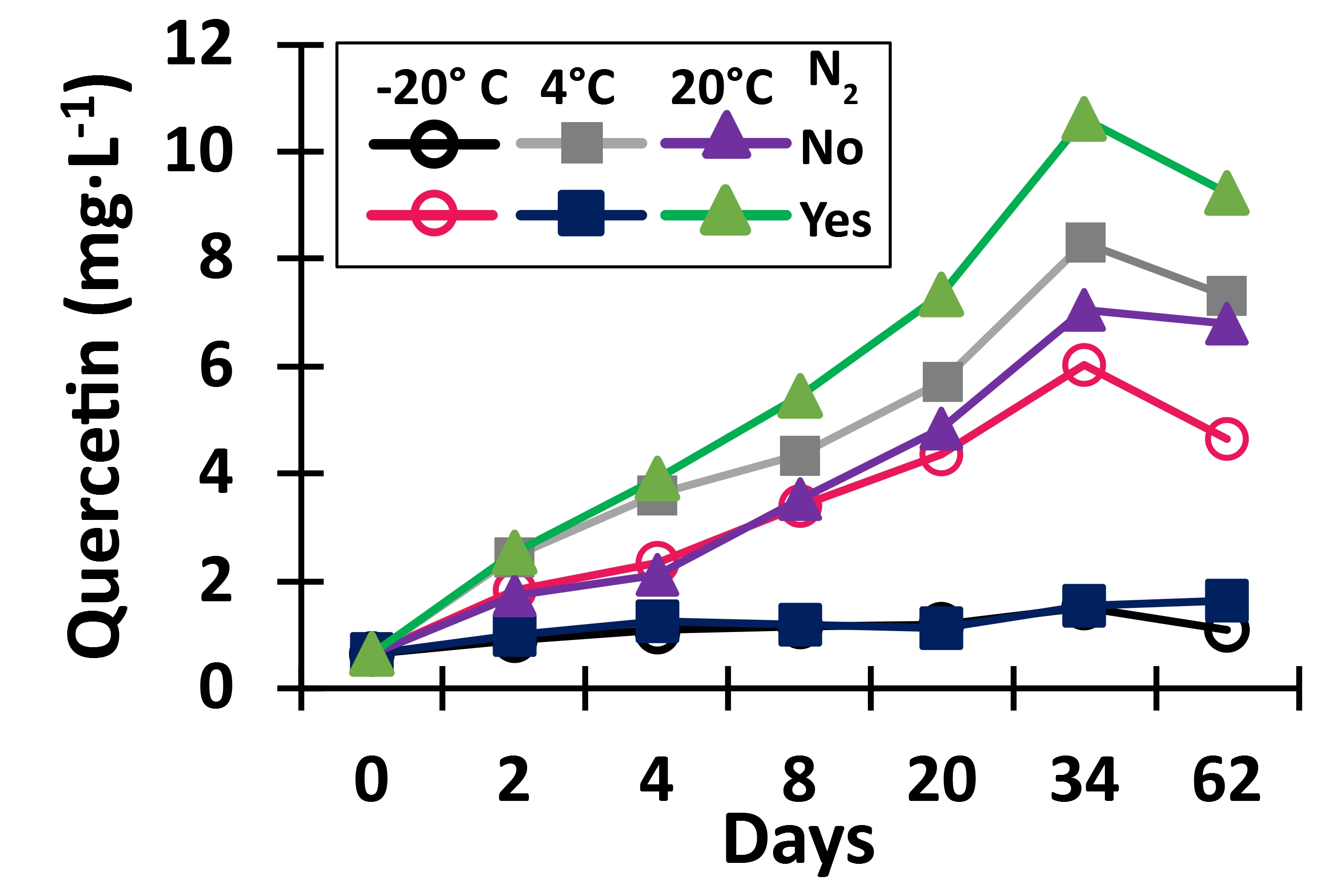 |
| **(G)** | **(H)** | **(I)** |
| 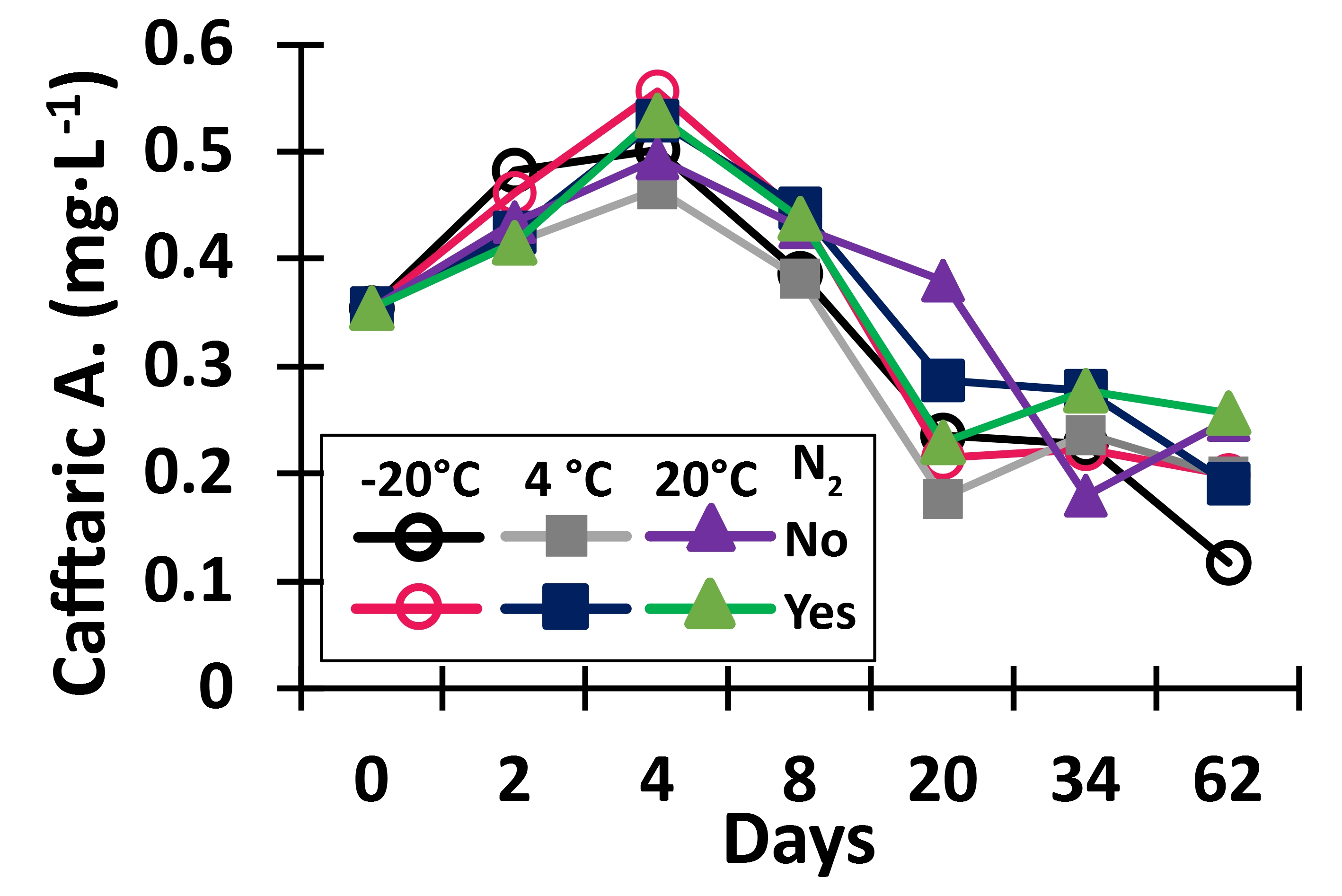 | 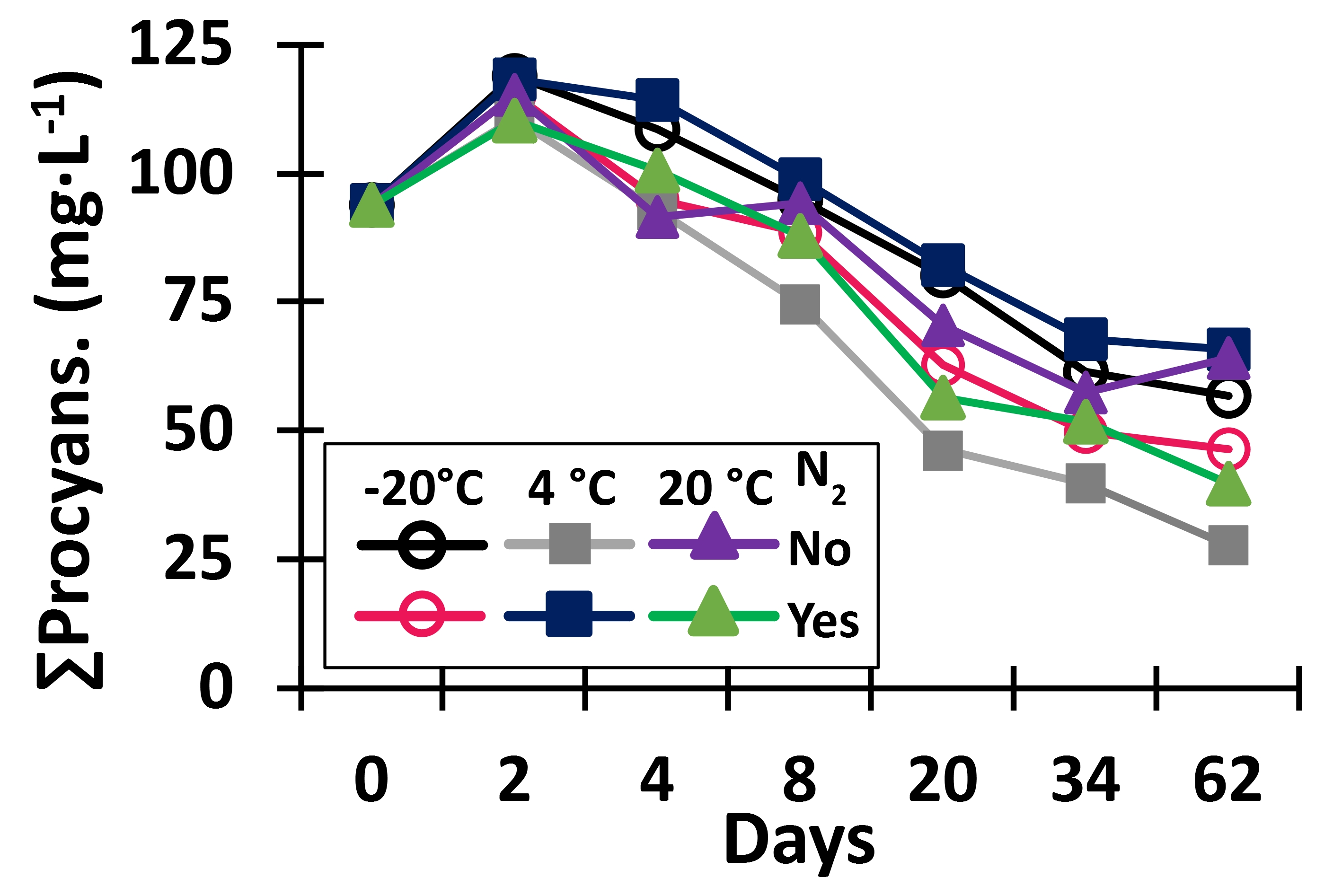 | 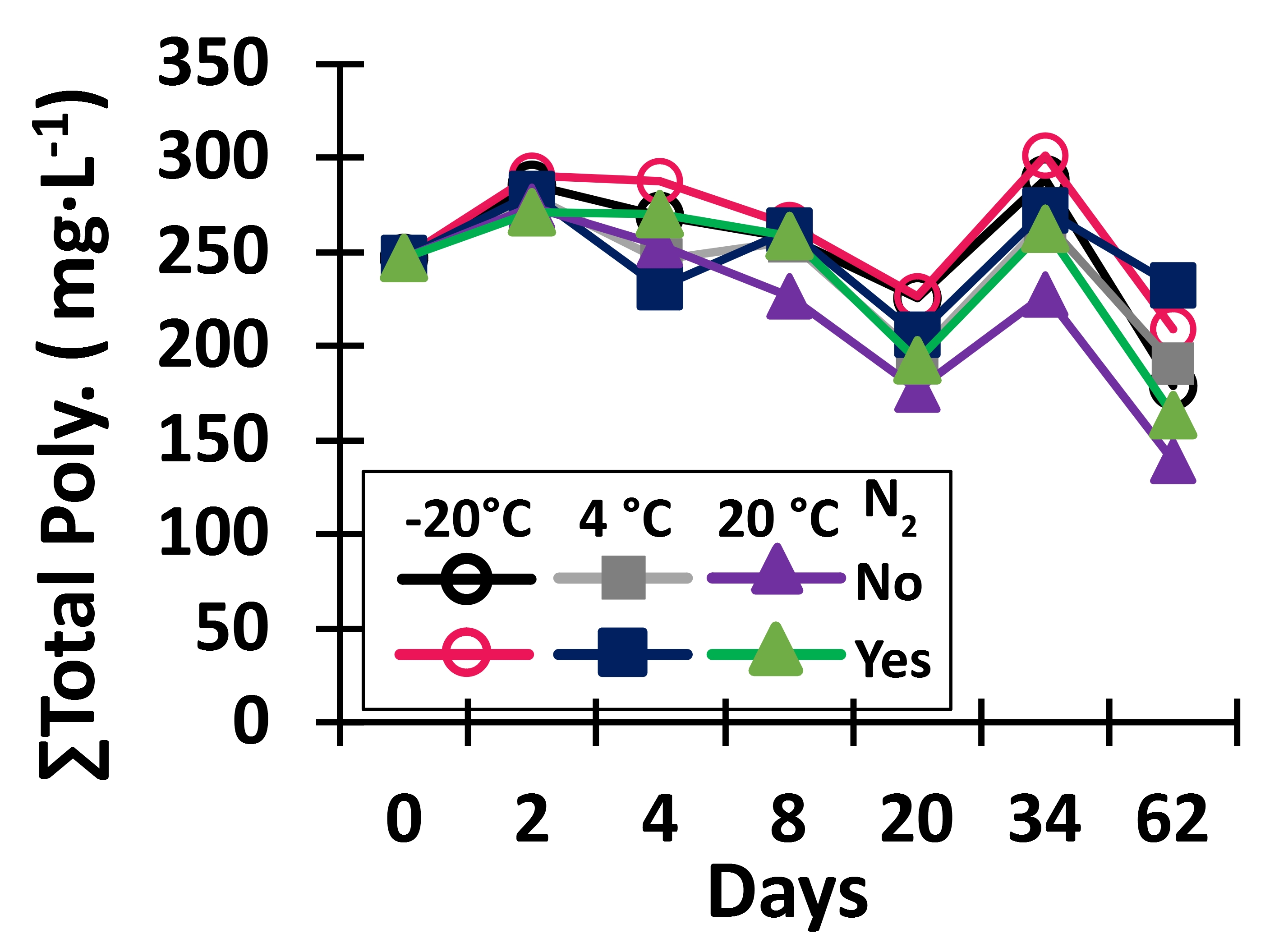 |
| **(J)** | **(K)** | **(L)** |

**Supplementary Figure 2.** Stability of the individual profile of the 13 polyphenolic compounds together with their total concentration present in the grape marc extract to changes in storage conditions (temperature and oxidative exposure) for 62 days of analysis. **(A)**. Gallic acid; **(B)**. Catechin; **(C)**. Epicatechin; **(D)**. Epigallocatechin gallate; **(E)**. Epicatechin gallate; **(F)**. Quercetin-3-glucuronide; **(G)**. Rutin; **(H)**. Quercetin-3-glucoside; **(I)**. Quercetin; **(J)**. Cafftaric Acid; **(K)**. Total procyanidins; **(L)**. Total polyphenols.
